# Networked SIRS model with Kalman filter state estimation for epidemic monitoring in Europe

**DOI:** 10.1038/s43856-026-01611-9

**Published:** 2026-05-08

**Authors:** Atte Aalto, Daniele Proverbio, Giulia Giordano, Alexander Skupin, Jorge Gonçalves

**Affiliations:** 1 Luxembourg Centre for Systems Biomedicine, University of Luxembourg, Belvaux, Luxembourg; 2 Department of Industrial Engineering, University of Trento, Trento, Italy; 3 Department of Physics and Material Sciences, University of Luxembourg, Belvaux, Luxembourg; 4 Department of Neurosciences, University of California, San Diego, CA USA; 5 Department of Plant Sciences, University of Cambridge, Cambridge, UK

**Keywords:** Epidemiology, Viral infection

## Abstract

**Background:**

Metapopulation models, which consider epidemic spread across interconnected regions, can provide more accurate epidemic predictions compared to isolated models for the corresponding regions. Still, their added complexity and data requirements raise questions about their tangible benefits over simpler, localized models.

**Methods:**

We develop and validate two networked compartmental metapopulation models for predicting influenza-like illness across Europe: a detailed network-based model, including international travel dynamics, and a simpler mean-field model, aggregating average regional data. The network is constructed using public mobility data and complemented with population densities at border regions. Incidence data of influenza-like illnesses from 28 countries are integrated using an Extended Kalman filter.

**Results:**

We show that networked epidemic models effectively capture epidemic dynamics across regions and epidemic phases. The models enable accurate forecasts, missing data imputation, and actionable insights: network models outperform isolated models in forecasting epidemic progression, particularly during critical periods such as wave onsets and peaks, and maintain reliability in scenarios with missing data.

**Conclusions:**

The findings unveil and quantify the advantages of metapopulation models for epidemic forecasting in interconnected regions, and pave the way to the integration of mobility and epidemic surveillance to improve the monitoring and prediction of spreading diseases.

## Plain language summary

Flu and other respiratory illnesses spread quickly across countries, especially in regions like Europe where people travel frequently. To better understand and predict these patterns, we built two mathematical models that predict how flu-like illnesses move between countries using information about travel dynamics and national illness reports. These models were tested using data from 28 European countries and found that they can track and forecast outbreaks more accurately than models that look at each country on its own. The models were especially good at predicting when new waves of illness would start and peak, and they could also fill in gaps when some countries had missing or delayed data. These results show that combining mobility and health data can improve how we monitor and anticipate disease spread, helping public health agencies respond faster and more effectively.

## Abstract (web summary)

Aalto et al. develop a pan European epidemiological network model to improve forecasting of influenza like illness across countries over a 10 year period. The network model outperforms single country models in forecast accuracy, in identifying the onset of epidemic waves, and in handling missing data.

## Introduction

Modern epidemiology increasingly relies on mathematical models to integrate empirical data, perform advanced analytics, and provide quantitative predictions that help forecast and control the spread of infectious diseases^1,2^. These models not only predict epidemic progression and future scenarios, but also support the development of early warning systems^3^, the assessment of the impact and effectiveness of restrictions^4^, and the estimation of unreported cases^5^ or cryptic transmission^6,7^. Hence, advancing forecasting methodologies is crucial to enable timely and effective public health responses and healthcare resource allocation, as testified by initiatives like Influcast^8^, Respicast^9,10^, and similar CDC initiatives^11^ aimed at providing platforms for integrating and benchmarking diverse forecasting models for influenza-like illnesses (ILI).

Traditional epidemiological models often focus on individual countries or regions, with the aim of predicting the overall spread of infectious diseases based on available data^12^, but without capturing the broader dynamics of interconnected regions. In contrast, metapopulation models^13–16^, based on the theory of epidemic spreading on complex networks^17–20^ and multi-patch mathematical models^21–23^, can predict the spatiotemporal progression of infectious diseases across large interconnected areas by incorporating mobility networks^24–26^ or distance-based networks^16^.

Recent studies have successfully employed network-based metapopulation models to forecast real-world epidemic dynamics, including COVID-19 across Italian regions^27,28^, influenza across U.S. states^29,30^, and dengue in China’s Guangdong province^31^. In fact, considering mobility at any given spatial granularity (e.g., between regions or states) has been shown to provide additional information compared to only considering region-wise incidence^30,32,33^. Despite the significant potential of metapopulation models, a critical question persists: given the same availability and spatial resolution of data for a given disease, do metapopulation models offer improvements in forecasting accuracy, and for which aspects of epidemic dynamics, compared to isolated models^34^?

Here we investigate whether a network-based metapopulation model improves epidemic forecasts for ILI over multiple seasons in Europe. Using empirical data on European mobility, we construct two models of increasing complexity: a mean-field model focuses on the epidemic evolution within a country and the average incidence in Europe, requiring relatively few data; a detailed network-based model accounts for inter-country travel dynamics and requires additional data on the network structure of mobility between countries. To estimate and predict disease dynamics, both models use an Extended Kalman Filter (EKF), an automated calibration method, also employed in alternative versions by earlier epidemiological studies^5,29–31,34–37^, to fruitfully integrate mechanistic models with empirical data and provide forecasts. The performance of the models is characterized by comparison against corresponding country-specific models, during 10 years of weekly ILI incidence data. These country-specific models were among the top-performing methods in the Respicast dashboard during the season 2023–2024^10^. Our results reveal and quantify the benefits of metapopulation models for epidemic forecasting in interconnected regions, offering probabilistic forecasts and scenario modeling at a continental scale. Our findings show that augmenting localized information with network-based data improves prediction accuracy, particularly for key periods such as wave onset, and resilience to missing data. Moreover, our framework can be adapted to other diseases and geographic scales, and thus offers a versatile tool for global public health applications.

## Methods

**Fig. 1:**
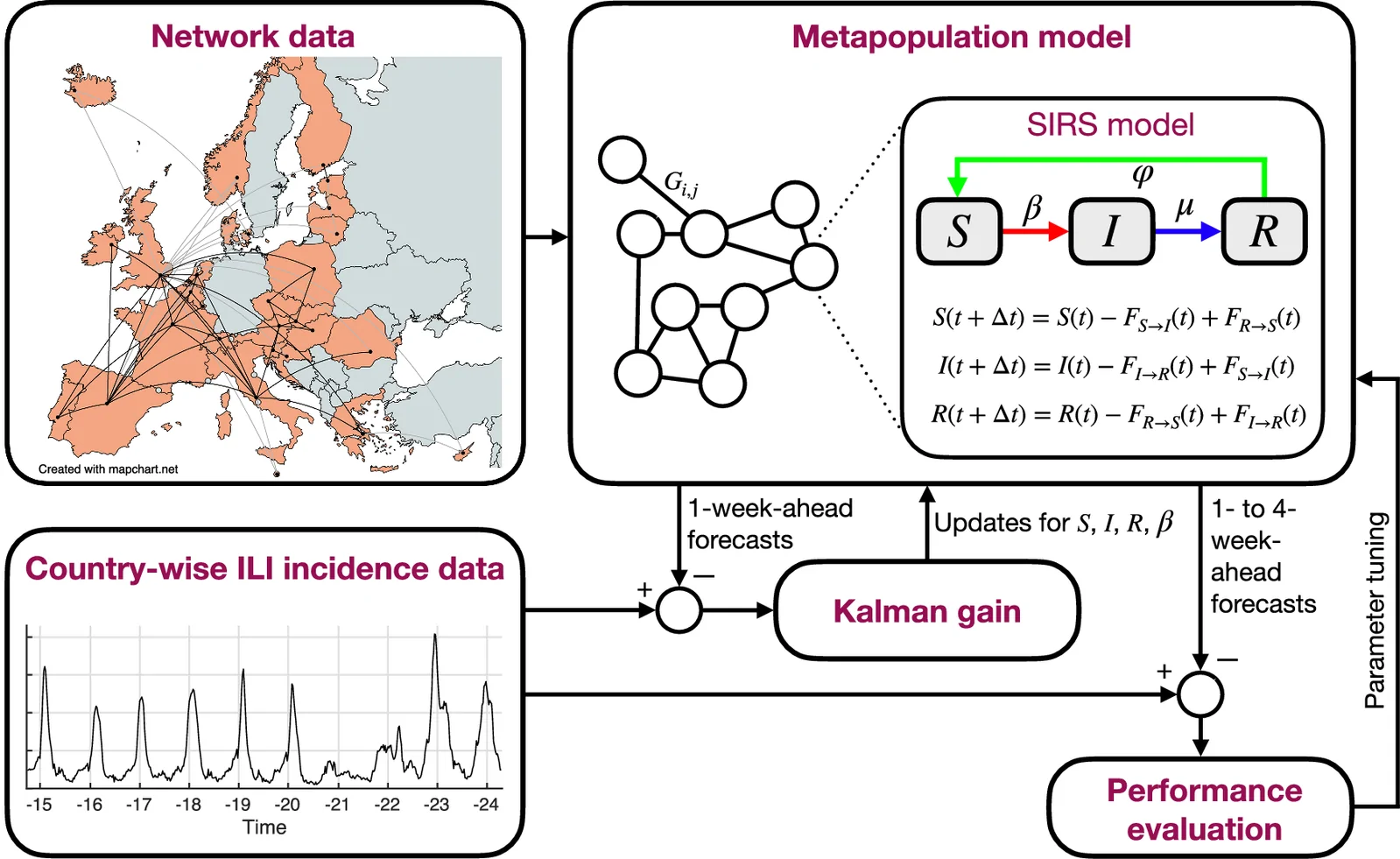
Model scheme. The network data panel shows daily fluxes above 10,000 with black lines, and the two strongest connections for each country with gray lines.

This section first outlines the construction of the mobility network; then, it introduces the dynamical model used to simulate the spread of ILI across 28 European countries. The model is based on a stochastic discrete-time Susceptible-Infectious-Removed-Susceptible (SIRS) framework, which describes the time evolution of the disease spread in homogeneous and well-mixed populations^38–41^. The model is extended to incorporate network effects to capture both local epidemic dynamics within each country and the influence of cross-border interactions. The full pipeline integrating the model with empirical data through an EKF is schematically represented in Fig. 1.

### Network generation

**Fig. 2:**
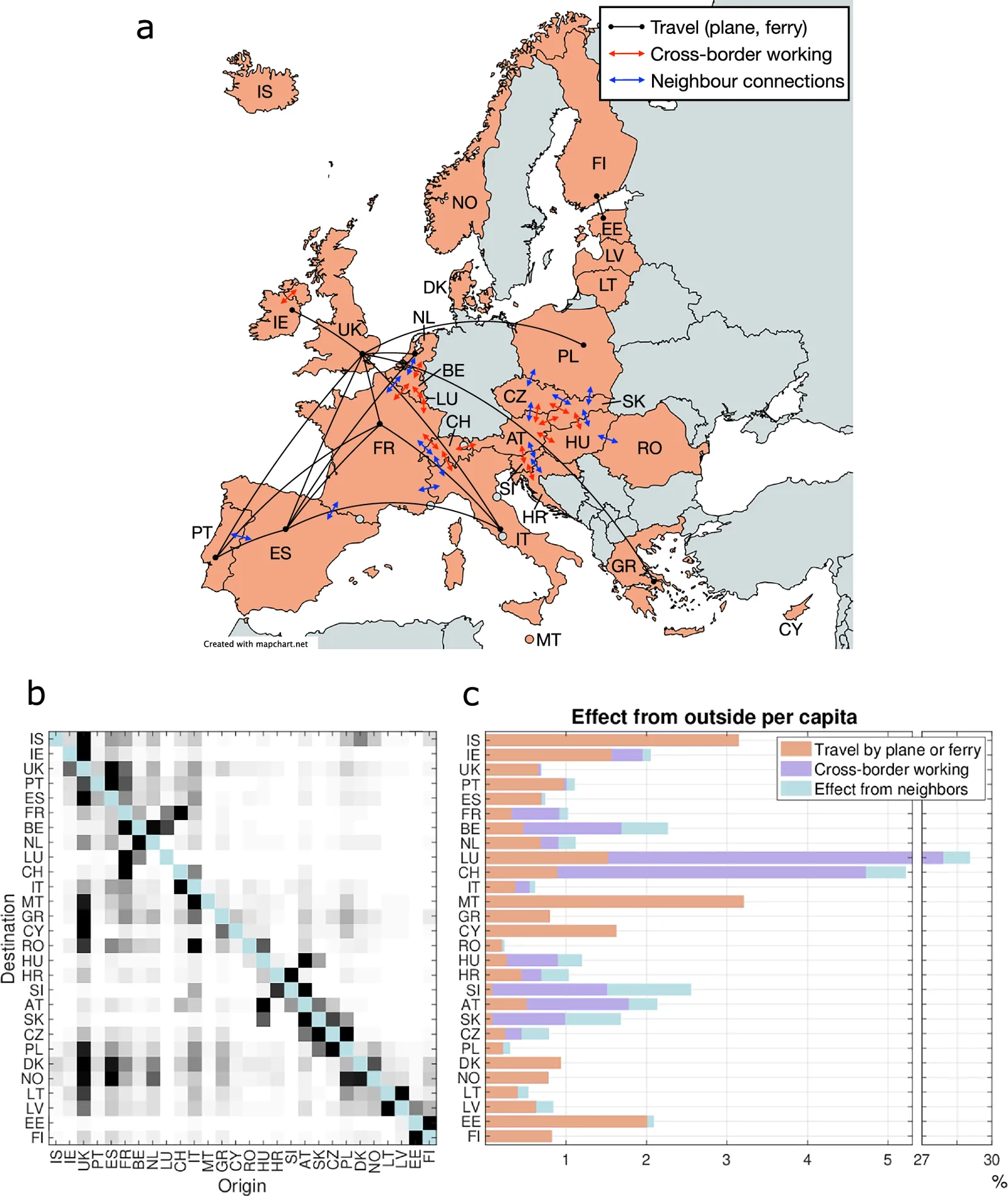
The constructed network. **a** The countries included in the network are highlighted in light red. The 15 busiest travel connections (by plane or ferry on any route between the indicated countries) are indicated with black lines (>10,000 passengers/day/direction). Red arrows show the 15 busiest cross-border worker flows (>3000 workers), and blue arrows show the 15 strongest neighbor connections of other types. **b** Visualization of the network matrix *G*. For visualization, each row has been normalized to have maximum value one. Darker color indicates stronger effect. **c** The effect from the outside for each country, calculated as the row sums of the network matrices *γ*_1_*G*^(1)^, *γ*_1_*G*^(2)^, and *γ*_2_*G*^(3)^ shown as percentages of the respective countries' populations. Only effects from other countries included in the network are considered.

To construct the metapopulation model, we first developed a mobility network representing population flows between European countries; see Fig. 2a. The network was built using publicly accessible data sources, ensuring consistency and comprehensive coverage. Alternatively, the network could be inferred from epidemic data, but this has been shown to be a severely ill-posed problem^42^. Each country was modeled as a node, with links representing three key types of travel flows: air and ferry travel, cross-border commuting, and other forms of land-based movement between neighboring countries. These components account for diverse modes of mobility, providing a realistic depiction of cross-border interactions. Here, we provide an overview of the network structure. An alternative to our travel-based network would be a distance-based network, such as the “gravity model”^16^. Supplementary Fig. 2 compares our network to the gravity network, highlighting that the gravity model underestimates connections based on popular flight (long-distance) destinations while overestimating mobility from certain large countries such as Italy or Poland. When possible, it is thus more effective to directly use empirical data to set the mobility matrix.

#### Travel network

Data for plane travels in 2023 were obtained from Eurostat^43^; only for UK, we used data from 2019 as the most recent available data before the COVID-19 pandemic. The flight data are given quarterly, and we averaged them over the whole year. Some missing value imputation is done based on data from different quarters between the countries in question, accounting for overall quarterly differences. Ferry traffic data were gathered from the statistics of the Finnish Port Association for Finland^44^ and from the UK Government, Department for Transport^45^. While COVID-19 disrupted air and ferry travel patterns, we used a fixed network for the entire period based on either pre- or post-pandemic data. Future studies may embed dynamical data flows, when available for the whole network.

#### Cross-border commuting network

Cross-border worker flows were extracted from reports by the European Commission’s Directorate-General for Employment, Social Affairs and Inclusion^46^ for the EU and EFTA areas, and the Economic and Social Research Institute^47^ for the UK-Ireland border. Commuter flows between non-neighboring regions were excluded, as they are typically captured within the air and ferry travel data. It was assumed that cross-border workers actually cross borders on 60% of days (thus excluding weekends, bank holidays and average leave periods). Cross-border workers were counted in the network as flows in both directions, between their country of residence and the country of work, as they were assumed to commute daily and thus be able to carry an infection in either direction.

#### Construction of neighbor graph

The neighbor graph lumps together all other travels across the land borders of different countries in the absence of available data. For each NUTS 3 region^48^ that shares a border with another country, we measured the length of the border segment between the region and the neighboring country, and weighted that with the population density of the region^49^. Weighted segments were then added up for the whole border between two countries, including the border regions of both countries so as to have a symmetric network matrix. The numbers obtained in this way are considered as dimensionless units and later scaled by parameter fitting. An example for this procedure for the Czechia–Slovakia border is shown in Supplementary Fig. 1.

This procedure provides a simple estimation of unregistered movements, but it has some shortcomings. For example, it looks at only one side of the border at a time. In reality, there is likely more traffic across the border if both sides of the border are densely populated. For example, Vienna and Bratislava, the capitals of Austria and Slovakia, are very close to each other. Although our procedure predicts fairly high traffic across the border, it might still be under-estimated. Another simplification is the omission of geographical specificities. For example, the borders between France and Italy and France and Spain are on mountainous regions. Even though both sides of these borders are densely populated, the traffic across these borders might be over-estimated by our procedure. Having better curated datasets may further improve this estimation in future applications.

Some outlier flows appear by this procedure. These outliers arise due to densely populated regions, with narrow or protruding shape, that share a relatively long border with a neighboring country. In addition, some cities constitute their own regions in the NUTS 3 classification, and if these cities lie at the border, they may cause outliers as well. These outliers are dealt with either by joining the corresponding regions with (larger) neighboring regions or, in few cases, by manually modifying the border length. Details on handling outliers are described in Supplementary Note 1.

#### Network structure and parameters

Our network matrix integrates the three mobility components, weighted by tuning parameters *γ*_1_ and *γ*_2_, to account for the relative contributions of different forms of travel. The population fluxes corresponding to air-and-ferry and commuting flows, denoted by matrices *G*^(1)^ and *G*^(2)^, were combined and scaled by a tuning parameter *γ*_1_ to account for varying durations of cross-border trips and for travel-associated infection risks, while the land-based movement component, denoted by *G*^(3)^, was scaled by a different tuning parameter *γ*_2_ since *G*^(3)^ does not readily correspond to actual fluxes and thus requires proper dimensionalization and rescaling. The resulting network matrix

1 $$G={\gamma }_{1}\left({G}^{(1)}+{G}^{(2)}\right)+{\gamma }_{2}{G}^{(3)}$$

captures the influence of mobility on epidemic dynamics, enabling detailed modeling of interconnected populations. The complete network is visualized in Fig. 2b as the heatmap of the row-wise normalized adjacency matrix ${G}_{i,j}/\max ({G}_{i,\cdot })$ between countries of origin and destination (normalization done only for visualization).

#### Regional impacts of the network

In almost all countries, the contribution of traveling people to the spread of ILI diseases is estimated to be less than 5% (Fig. 2c). An exception is Luxembourg, where cross-border workers represent an important fraction of the effective population. For island countries, or those where the major urban areas are close to shores (e.g., Iceland, UK, or Finland), the largest effect is due to air and ferry travel.

By combining diverse mobility data sources into a unified framework, our network generation approach provides a foundational framework to simulate disease spread across Europe. The methodology is flexible and can be extended to other countries or regions as new data become available.

#### Mean-field approximation

To explore the trade-off between data availability and model complexity, we implemented a simpler “mean-field” version of the model, where the detailed network matrix *G* is replaced with

2 $${\widehat{G}}_{i,j}=\gamma \frac{{N}_{i}{N}_{j}}{{\sum }_{k=1}^{28}{N}_{k}},$$

where *γ* is a tuning parameter and *N*_*i*_ is the population of country *i*. This approach is less granular, but requires fewer data and is suitable for scenarios with incomplete mobility information. The heatmap for the mean-field network matrix is shown in Supplementary Fig. 2.

#### Individual models

The performances of the network-based and mean-field models were compared against country-specific isolated models that do not consider cross-border interactions, that is, such models do not account for a network matrix *G*.

### SIRS model at each node of the network

To simulate disease dynamics at each node of the network, we employ a discrete-time SIRS model:

3 $$\begin{array}{l}S(t+\Delta t)=S(t)-{F}_{S\to I}(t)+{F}_{R\to S}(t)\\ I(t+\Delta t)=I(t)+{F}_{S\to I}(t)-{F}_{I\to R}(t)\\ R(t+\Delta t)=R(t)+{F}_{I\to R}(t)-{F}_{R\to S}(t)\end{array}$$

where *F*_*X*→*Y*_(*t*) is a shorthand notation for the flow from compartment *X* to compartment *Y* at time *t*, with unit of 1 week (we will later omit the time-dependency). The model’s time step is 1 day, *Δt* = 1/7. The model satisfies the conservation law

$$S(t+\Delta t)+I(t+\Delta t)+R(t+\Delta t)=S(t)+I(t)+R(t).$$

In the country-specific SIRS model, the fluxes between compartments are given by *F*_*S*→*I*_(*t*) = *β*(*t*)*S*(*t*)*I*(*t*)/*N*, *F*_*I*→*R*_(*t*) = *μI*(*t*), and *F*_*R*→*S*_ = *φR*(*t*) where *μ* = 0.06 and φ=ln(2)/60=0.0116. Parameter *μ* is set to a small value because, to guarantee stability of the state estimation, the time scale of the model dynamics should be close to the time scale of data collection. Parameter *φ* corresponds to immunity half life of 60 days: ILI may be caused by many different viruses, with varying cross-immunity properties, so this parameter should be considered as an effective parameter rather than a reflection of the human immune system. In the metapopulation network model, *M* countries with populations *N*_*i*_ (*i* = 1…*M*) are represented as nodes in the network, with epidemic dynamics described by SIRS equations (3). The regions are connected by the mobility network matrix *G* defined in (1). Its entries *G*_*i*,*j*_ represent effective flows of individuals from region *j* to region *i* per day. The flows are called “effective” since we will estimate global parameters *γ*_1_ and *γ*_2_ in (1) multiplying the network matrix based on forecasting performance. In the network model, the number of new infections in country *i* in 1 day, that is, the flux from the susceptible population *S*_*i*_(*t*) to the infectious population *I*_*i*_(*t*) at time *t*, is given by

4 $${F}_{{S}_{i}\to {I}_{i}}(t)=\frac{{\beta }_{i}(t){S}_{i}(t)}{{N}_{i}}\left({I}_{i}(t)+{\sum }_{j=1}^{M}{G}_{i,j}\frac{{I}_{j}(t)}{{N}_{j}}\right).$$

The flux depends on the infectious population within the country *i* itself, but also other countries: *I*_*j*_(*t*)/*N*_*j*_ is the fraction of infected individuals in country *j*; hence assuming perfect mixing of populations, the flux of infected individuals from country *j* to country *i* is *G*_*i*,*j*_*I*_*j*_(*t*)/*N*_*j*_. We assume that these flows are symmetric: *G*_*i*,*j*_ = *G*_*j*,*i*_. This assumption is a slight simplification of a more general conservation law stating that the population of each country remains constant, which would require ${\sum }_{i}{G}_{j,i}={\sum }_{i}{G}_{i,j}$ for all *j*. We do not differentiate between people traveling to another region and becoming infected there, and infectious people traveling and infecting others at the destination region.

For the mean-field network model, instead, we substitute *G* defined in Eq. (2) into (4) to get

5 $${\widehat{F}}_{{S}_{i}\to {I}_{i}}(t)={\beta }_{i}(t){S}_{i}(t)\left(\frac{{I}_{i}(t)}{{N}_{i}}+\gamma \frac{{\sum }_{j=1}^{M}{I}_{j}(t)}{{\sum }_{j=1}^{M}{N}_{j}}\right)$$

where the second term inside the parentheses does not depend on the target country *i*, but is the average prevalence over all included countries, modulated by the parameter *γ*. This model ignores geographical and other detailed information on the traffic between countries. For the country-specific models, we set *G*_*i*,*j*_ = 0 to recover the isolated node-specific SIRS model.

For all models, the other fluxes *F*_*I*→*R*_(*t*) and *F*_*R*→*S*_(*t*) are as in the isolated models, and do not assume dependency on travels.

### Stochastic model

Towards an implementation of the EKF, a stochastic version of the networked SIRS model (4) is obtained based on the method described in ref. ^37^. We follow the derivation of a Langevin equation^50^, that is, we pose that each susceptible person has a probability (in the non-networked version, for simplicity) *β*(*t*)*I*(*t*)/*N* to become infected at time *t*. The number of new infections on a day at time *t* is then binomially distributed with mean $\frac{\beta (t)I(t)}{N}S(t)$ and variance $\frac{\beta (t)I(t)}{N}\left(1-\frac{\beta (t)I(t)}{N}\right)S(t)$. Assuming $\frac{\beta (t)I(t)}{N}\ll 1$, the term with ${\left(\frac{\beta (t)I(t)}{N}\right)}^{2}$ is omitted from the variance. Further assuming $\frac{\beta (t)I(t)}{N}S(t)$ is large enough, the binomial distribution can be well approximated by the normal distribution with the same mean and variance.

As a result, each transition between compartments is accompanied by a stochastic white noise process modulated by a multiplicative state-dependent term, which scales as the square root of the transition rate. However, the derivation of the stochastic model implicitly assumes a homogeneous and well-mixed population. This assumption leads to severely underestimated noise levels. To account for this underestimation, the variance terms are multiplied by a tuning factor *κ*_1_. In addition, a second term ${\kappa }_{2}{N}_{i}^{2}$ is added to the variance of the transition from *S* to *I*, that does not depend on the epidemic state. This can be interpreted as import of cases from countries that are not included in the network; a second reason behind its introduction is technical: during summer periods, many countries stop the ILI monitoring and reporting, hence the model tends to a state where there are no new cases emerging. Then, also the uncertainty on the epidemic state eventually converges to zero. When reporting is restarted, the Kalman filter can mainly make updates on the *β*-parameter due to its unfounded certainty on the model variables. This leads to poor projections in the beginning of a new epidemic season. The term ${\kappa }_{2}{N}_{i}^{2}$ is introduced to mitigate this problem by providing inertia to uncertainties at the beginning of a new season. The final stochastic networked SIRS model is:

6 $$S(t+\Delta t)= 	 \, S(t)-{F}_{S\to I}+{F}_{R\to S}-\sqrt{{\kappa }_{1}{F}_{S\to I}}{w}_{1}(t)+\sqrt{{\kappa }_{1}{F}_{R\to S}}{w}_{3}(t)-\sqrt{{\kappa }_{2}}N{w}_{4}(t)\\ I (t+\Delta t)= 	 \, I(t)+{F}_{S\to I} -{F}_{I\to R}+\sqrt{{\kappa }_{1}{F}_{S\to I}}{w}_{1}(t)-\sqrt{{\kappa }_{1}{F}_{I\to R}}{w}_{2}(t) +\sqrt{{\kappa }_{2}}N{w}_{4}(t) \\ R(t+\Delta t) = 	 \, R(t)+{F}_{I\to R}-{F}_{R\to S} +\sqrt{{\kappa }_{1}{F}_{I\to R}}{w}_{2}(t)-\sqrt{{\kappa }_{1}{F}_{R\to S}}{w}_{3}(t)$$

where *w*_*k*_(*t*) are mutually independent Gaussian white noise processes with unit variance.

### Dynamics of transmission parameter

The transmission parameters *β*_*i*_(*t*) in (4) and (5) are modeled as time-varying variables influenced by both local and network-level factors. Changes in *β*_*i*_(*t*) can reflect shifts in population behavior, interventions, seasonal effects, or varying transmissibility of different viral strains. The dynamics of *β*_*i*_(*t*) are governed by

7 $${\beta }_{i}(t+\Delta t)={\beta }_{i}(t)+{k}_{1}\left[{k}_{2}\frac{{\sum }_{j}{G}_{i,j}{\beta }_{j}(t)}{{\sum }_{j}{G}_{i,j}}+(1-{k}_{2}){k}_{3}\mu -{\beta }_{i}(t)\right]+{w}_{\beta }(t),$$

where *w*_*β*_(*t*) is Gaussian noise with constant variance *σ*_*β*_ and the term inside the brackets acts as a nudge towards a reference value that is a combination of the weighted average of *β*_*j*_’s in the neighbors of country *i* and a “grounding value” *k*_3_*μ* corresponding to a basic reproduction number *R*_0_ = *k*_3_. The tuning parameters *k*_1_, *k*_2_, and *k*_3_ all have their own roles: *k*_1_ modulates the strength of the nudging towards the reference value, *k*_2_ is the weight between the two components of the reference value, and *k*_3_ scales the grounding value. In isolated models, *k*_2_ is zero. In the absence of data, the *β*-parameters tend to the grounding value *k*_3_*μ*, and, consequently, the system would eventually converge to an endemic steady state, which is (for the isolated models) *S* = *N*/*k*_3_, $I=\frac{\phi ({k}_{3}-1)}{(\phi +\mu ){k}_{3}}N$, and $R=\frac{\mu ({k}_{3}-1)}{(\phi +\mu ){k}_{3}}N$.

In contrast to the model in ref. ^37^, we include deterministic dynamics for the *β*-parameters. The reason for the inclusion is threefold. Firstly, due to missing data in many countries during the summer periods, the Kalman filter does not make significant updates to the *β*_*i*_ parameters during these periods. However, it is reasonable to assume that, before seasonal re-emergence of ILI, the effective transmission approaches similar values each year, without retaining the value reached at the end of the previous wave. The deterministic dynamics for *β*_*i*_ first takes into account this seasonality. Secondly, some effects modeled by the time-varying *β*-parameters are not country-specific, such as seasonal effects and transmissibility of a prevailing viral strain. Thirdly, by allowing the country-specific *β*_*i*_ to be influenced by the parameters of the neighbors in the network, the parameter estimates become more stable. This inclusion of network effects in the *β*-parameter dynamics differs significantly from previous models^30,31,34^ as it allows regional waves to influence neighboring countries, thus enhancing the model’s ability to anticipate epidemic patterns (as seen in Supplementary Fig. 7).

### Extended Kalman filter implementation

**Fig. 3:**
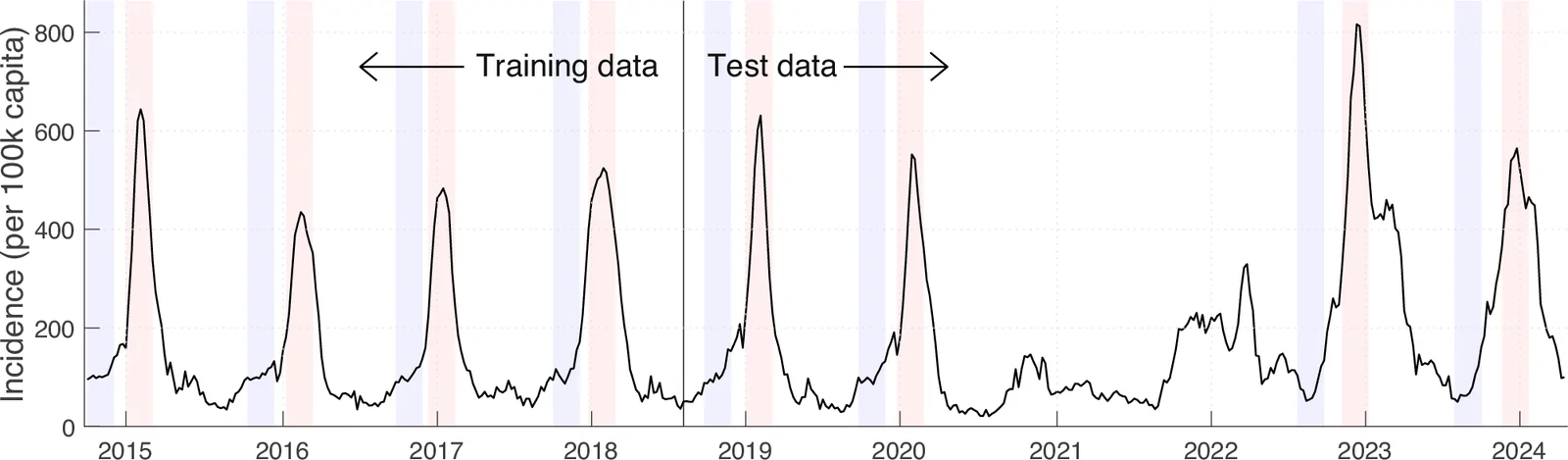
Average ILI incidence across Europe. Influenza-like illness (ILI) incidence data were obtained from the “Respicast” project^9^, which consolidates data from the ECDC's European Respiratory Virus Surveillance Summary (ERVISS)^53^ and the World Health Organization’s FluID global influenza program^54^. The dataset spans ten epidemic seasons (2014–2024). The figure shows the population-weighted average ILI incidence across Europe. Blue-shaded areas indicate wave onset periods, while red-shaded areas mark epidemic peaks. These critical periods were used for evaluating forecasting performance in subsequent analyses. The data were aggregated from weekly reports and adjusted for population differences across reporting countries. The figure highlights the seasonal ILI dynamics and the variation in timing and intensity of epidemic waves.

To estimate epidemic parameters and make accurate predictions, the network-based SIRS model was coupled with an EKF^51,52^. The EKF integrates real-world observations with the model dynamics, providing robust state estimation and allowing for dynamic adjustments as new data become available. The EKF requires an underlying dynamical model (in our case, the stochastic networked SIRS model), its output, covariance matrices for state noise and measurement noise, and measurement data (Fig. 3), provided as weekly aggregated case numbers. The EKF works recursively involving a prediction step using the model, and then an update of the state vector based on discrepancies between predictions and observations, ensuring that the model evolution is close to real-world dynamics. The algorithm is presented in Supplementary Note 2.

To apply the EKF into the networked system, the state of the system for each country is represented with a 3-dimensional state vector, consisting of *S*_*i*_(*t*), *I*_*i*_(*t*), and *β*_*i*_(*t*). The *R*_*i*_(*t*) compartments are not included, since they can be recovered using the conservation law, *R*_*i*_(*t*) = *N*_*i*_ − *S*_*i*_(*t*) − *I*_*i*_(*t*). The 3-dimensional state vectors for all 28 countries are concatenated into one 84-dimensional state vector *x*, for which we have a stochastic model

x(t+Δt)=f(x(t))+W(t).

The function $f:{{\mathbb{R}}}^{84}\to {{\mathbb{R}}}^{84}$ is defined block-wise $f(x)={[{f}_{1}(x),\ldots ,{f}_{28}(x)]}^{\top }$ country by country through equations (3), (4), and (7). The state noise vector *W*(*t*) includes the contributions of all noise terms in the stochastic model (6), and from the stochastic model we can obtain its covariance matrix *Q* as a block diagonal matrix whose *i*^th^ block is given by

8 $${Q}_{i}=\left[\begin{array}{ccc}{\kappa }_{1}({F}_{{S}_{i}\to {I}_{i}}+{F}_{{R}_{i}\to {S}_{i}})+{\kappa }_{2}{N}_{i}^{2} & -{\kappa }_{1}{F}_{{S}_{i}\to {I}_{i}}-{\kappa }_{2}{N}_{i}^{2} & 0\\ -{\kappa }_{1}{F}_{{S}_{i}\to {I}_{i}}-{\kappa }_{2}{N}_{i}^{2} & {\kappa }_{1}({F}_{{S}_{i}\to {I}_{i}}+{F}_{{I}_{i}\to {R}_{i}})+{\kappa }_{2}{N}_{i}^{2} & 0\\ 0 & 0 & {\sigma }_{\beta }\end{array}\right].$$

The observed case numbers are also assumed to be affected by uncertainties. We assume that every infectious person has a probability *c*_*i*_(*t*) to be detected at week *t* and the detections are independent. Then, the number of detected cases is binomially distributed with mean *c*_*i*_(*t*)*I*_*i*_(*t*) and variance (1 − *c*_*i*_(*t*))*c*_*i*_(*t*)*I*_*i*_(*t*). The model-predicted number of detected cases for country *i* is therefore given by

9 $${\widehat{y}}_{i}(t)={c}_{i}(t){I}_{i}(t)$$

and the full observation vector $\widehat{y}(t)={[{\widehat{y}}_{1}(t),\ldots ,{\widehat{y}}_{28}(t)]}^{\top }$ is obtained as $\widehat{y}(t)=C(t)x(t)$ where *C*(*t*) is a 28 × 84 matrix whose element (*i*, 3(*i* − 1) + 2) is *c*_*i*_(*t*) with zeros elsewhere. The measurement noise covariance matrix *U* for the full observation vector is diagonal with elements

10 $${U}_{i}(t)=\left({\rho }_{1}(1-{c}_{i}(t)){y}_{i}(t)+{\rho }_{2}{N}_{i}\right){u}_{i}$$

where the first term inside the parentheses corresponds to the variance of the binomial distribution (using the observed number *y*_*i*_(*t*) as a proxy for *c*_*i*_(*t*)*I*_*i*_(*t*)). As we did with the stochasticity of new infections, here we also introduce a second, state-independent noise term whose variance is proportional to the population size *N*_*i*_ to enforce a lower bound to the variance during times of low incidence. *ρ*_1_ and *ρ*_2_ are tuning parameters for the different noise terms, and *u*_*i*_ is a country-specific coefficient measuring the noise level in the data, defined by

$${u}_{i}=\frac{1}{499}{\sum }_{t=1}^{499}\frac{{({y}_{i}(t)-{\bar{y}}_{i}(t))}^{2}}{{\bar{y}}_{i}(t)},$$

where ${{\bar{y}}}_{i}(t)$ denotes the 5-week moving average of *y*_*i*_, with the moving window centered at *t*. The coefficient *u*_*i*_ reflects country-specific differences in data collection and reporting practices. It compares the data variance over the entire dataset covering 499 weeks around a moving average to the variance of the binomial distribution, which is approximated by the moving average.

The parameter *c*_*i*_(*t*) in the model output adjusts for under-reporting. The ratio of detected and total cases is captured by $\frac{{c}_{i}(t)\Delta t}{\mu }$. In SIR-type models, the initial exponential growth rate of a new wave and the herd immunity level are tightly connected. However, if only a fraction of the infections are detected, this has no effect on the observed initial exponential growth rate but it directly affects the amplitude of the upcoming (detected) wave. Without knowledge of this fraction, the link between initial growth rate and wave amplitude is broken, which precludes (model-based) projections going beyond short-term extrapolation. Therefore, the *c*_*i*_(*t*) parameters are crucial for modeling the wave amplitude, and we have developed an adaptive scheme for their estimation.

As a small remark, note that *I*_*i*_(*t*) in (9) corresponds to ILI prevalence, whereas the data are ILI incidences. In the CoWWAn^37^ model and for the Respicast projections (during epidemic season 2023–2024), we used a slightly more complex observation model with an additional “counter” variable for the daily or weekly new cases precisely corresponding to disease incidence. However, this model implicitly assumed that all observations were from infections occurring on the same day or week, whereas the current model (9), where case numbers are simply obtained from the size of the *I*-compartment, assumes that an infection can be detected and counted at any time when the infected individual is in the *I*-compartment. We observed that forecasting performance was just as good with this simpler model warranting its implementation here.

After every Kalman update, the model (without noise) is run 4 weeks forward in time to produce incidence projections for *τ* = 1, 2, 3, 4 weeks ahead, denoted by ${\widehat{y}}_{i}(t+\tau | t)$. That is, ${\widehat{y}}_{i}(t+\tau | t)$ is a projection for week *t* + *τ* done using data up to week *t*.

### Adaptive estimation of *c*_*i*_(*t*)

#### Initial values

The initial values for the ratios are based on the observed epidemic dynamics for the first two epidemic waves in the data. The discounted case numbers are calculated as ${Y}_{i}(t;1-\nu )={\sum }_{\tau =0}^{t}{(1-\nu )}^{t-\tau }{y}_{i}(\tau )$ where $\nu =\frac{1}{1/\mu +1/\phi }$ represents the average flow rate through the *I* and *R* compartments (that is, 1/*ν* is the average time from infection to becoming susceptible again). Then

11 $${c}_{i}(1)={\alpha }_{1}\frac{{\max }_{t\in [{T}_{0,i},{T}_{0,i}+103]}{Y}_{i}(t;1-\nu )}{{N}_{i}}$$

where *T*_0,*i*_ denotes the first week when data are available for country *i*. Here *α*_1_ is a tuning parameter.

#### Upward update

If a too small value *c*_*i*_(*t*) is used for a country during an epidemic wave, the *S*-compartment will become exhausted. When this happens, more precisely, when *S*_*i*_(*t*) < 0.4*N*_*i*_ for some country, this is deemed unrealistic, and a parameter adaptation is triggered. The parameter *c*_*i*_(*t*) is retroactively increased, and the last 12 weeks are re-run with the higher parameter value. Further upward updates are not allowed during the re-run weeks.

The coefficient used for increasing *c*_*i*_(*t*) is determined as follows. The discounted true case numbers and 1-week ahead forecasts are calculated:

$${Y}_{i}(t;0.8)={\sum }_{\tau =1}^{t}0.{8}^{t-\tau }{y}_{i}(\tau ),\,{\widehat{Y}}_{i}(t;0.8)={\sum }_{\tau =1}^{t}0.{8}^{t-\tau }{\widehat{y}}_{i}(\tau ).$$

The parameter *c*_*i*_(*t*) is then increased by multiplying it with the coefficient

12 $$\max \left\{1.1,{\left(\frac{{Y}_{i}(t;0.8)}{{\widehat{Y}}_{i}(t;0.8)}\right)}^{{\alpha }_{2}}\right\}$$

where the exponent *α*_2_ is a tuning parameter used to link case numbers with the parameter *c*_*i*_(*t*). The parameter is increased retroactively in a piecewise linear fashion, that is, *c*_*i*_ has a linearly increasing phase from *t* − 12 to *t* − 6 from the old value to the increased value. From week *t* − 5 to *t* + 15, the parameter remains at the new higher value. From week *t* + 16 to *t* + 26 the parameter linearly decreases to a value that is *c*_*i*_(*t* − 12) + *α*_3_(*c*_*i*_(*t*) − *c*_*i*_(*t* − 12)), that is, *α*_3_ ∈ (0, 1) denotes a share of the parameter update that is kept for the next epidemic wave. This parameter is used only when an upward update is triggered; we observed very few triggering events during the training data period. Hence, *α*_3_ was estimated directly from data as illustrated in Supplementary Fig. 3.

Once the parameter has been updated, the last 12 weeks are re-run with the new parameter value. Note that the *τ*-week ahead projections $\widehat{y}(t+\tau | t)$ are only generated when week *t* is passed for the first time in the algorithm to ensure causality of the projections.

#### Downward update

Unlike for too small *c*_*i*_(*t*), there are no clear signs for too high value, except for overshooting projections, in particular, during times of high incidence. Therefore, downward updates are only done on pre-determined times between epidemic waves (that is, in summer). At such time *T*, the performance for the previous year is analyzed for all countries. To check for overshooting projections, for each country, we find the weeks from the previous year *T* − 51, …, *T* during which the incidence has been on the highest 30th percentile during that year (denote this set of weeks by **T**_*i*_(*T*)). The *c*_*i*_(*t*) is then reduced by multiplication by a factor

13 $$\min \left\{1,{\left(\frac{{\sum }_{t\in {{{{\bf{T}}}}}_{i}(T)}{\sum }_{\tau =1}^{4}{y}_{i}(t+\tau )}{{\sum }_{t\in {{{{\bf{T}}}}}_{i}(T)}{\sum }_{\tau =1}^{4}{\widehat{y}}_{i}(t+\tau | t)}\right)}^{{\alpha }_{2}}\right\}.$$

The reduction is done in a piecewise linear way, such that the linear descent takes place on weeks *T* + 1 to *T* + 6.

### Statistics and reproducibility

Travel data to construct the network model have been sourced from public mobility repositories or estimated using population census, as described in the section “Network generation”.

ILI incidence data originates from the ECDC’s European Respiratory Virus Surveillance Summary (ERVISS)^53^ and the World Health Organization’s (WHO) FluID global influenza program^54^. Since the study is based on publicly available and fully anonymized aggregate data, and because ILI does not constitute a sensitive topic, an ethical approval was not required^55^. The dataset spans ten epidemic seasons, from week 40 of 2014 to week 16 of 2024. Incidences are reported as weekly cases per 100,000 inhabitants. To enable integration into the metapopulation model, these incidences were scaled to estimate the actual number of detected infections for each country. For the United Kingdom, data were aggregated from England, Wales, Scotland, and Northern Ireland to align with available mobility data, which were reported for the whole UK.

All data were processed using the model-based approach described above, integrating them into the EKF, informed by stochastic dynamical epidemic models. The model parameters and the noise content are fitted and calibrated as described below. The complete pipeline is available on the project Gitlab repository (see “Data and Code availability”).

### Performance evaluation

For performance evaluation, we define the *τ*-week ahead forecasts ${\widehat{y}}_{i}(t+\tau | t)={c}_{i}(t+\tau ){\widehat{I}}_{i}(t+\tau | t)$ where ${\widehat{I}}_{i}(t+\tau | t)$ is obtained by simulating the noise-free model forward in time for *τ* weeks, starting from the Kalman filter state estimate after the update on week *t*.

The total forecasting error is obtained by summing up country-wise errors over all the 28 considered countries:

14 $$E({{{\bf{T}}}})	={\sum }_{i=1}^{28}{E}_{i}({{{\bf{T}}}}),\,{{{\rm{where}}}}\,{E}_{i}({{{\bf{T}}}})\\ 	={\sum }_{\tau =1}^{4}{\sum }_{t+\tau \in {{{\bf{T}}}}}\left|\sqrt{{y}_{i}(t+\tau )}-\sqrt{{\widehat{y}}_{i}(t+\tau | t)}\right|.$$

Here, **T** denotes the time interval or a collection of time intervals used for evaluation. The square root is used as transformation to stabilize variance, so as to balance the performance metric between times of high and low incidence, and between countries with larger and smaller population size. Note that weeks *t* where data for country *i* are missing for weeks *t* − 3, …, *t*, are excluded from *E*_*i*_.

As a reference to benchmark our predictions, we simply use the latest data point as a forecast for the next 4 weeks:

$${E}_{i}^{({{{\rm{ref}}}})}({{{\bf{T}}}})={\sum }_{\tau =1}^{4}{\sum }_{t+\tau \in {{{\bf{T}}}}}\left|\sqrt{{y}_{i}(t+\tau )}-\sqrt{\bar{y}_{i}(t)}\right|$$

where ${\bar{y}}_{i}(t)={y}_{i}(t)$ if these data exist, or equals the value of the previous existing data point, if *y*_*i*_(*t*) does not exist. We measure how much all different models, used for evaluation, improve the overall predictions w.r.t. the reference forecast, that is,

15 $$\,{{{\rm{Performance}}}}\; {{{\rm{gain}}}}\,=1-\frac{{\sum }_{i=1}^{28}{E}_{i}({{{\bf{T}}}})}{{\sum }_{i=1}^{28}{E}_{i}^{({{{\rm{ref}}}})}({{{\bf{T}}}})}\,,$$

calculated separately for the training data period and the test data period. In addition, to evaluate performance during the wave onset and peak periods, the errors *E*_*j*_ are collected over time intervals covering the onsets of all epidemic waves (except for 2020–2022, which were heavily affected by the COVID-19 pandemic). Here, we no longer considered the split between training and testing data. These time intervals were determined by identifying weeks *W* when the European-wide incidence crossed 120, and taking the interval from *W* − 8 to *W* + 1. Additionally, we collect all time intervals around the peak incidence, identified by weeks $\widehat{W}$ with the highest incidence during each epidemic season, and take the interval $\widehat{W}-5$ to $\widehat{W}+4$ (see Fig. 3).

To evaluate probabilistic forecasts, 1–4-week ahead forecasts were generated every other week. They were evaluated using the weighted interval score (WIS), commonly used to evaluate probabilistic forecasts in epidemiology^8,9,56^, that measures both the accuracy and confidence on the forecasts by penalizing for the width of the obtained confidence intervals. Since the WIS scales directly with the scale of the data, we normalize the scores for each country by the average peak incidence of the epidemic waves in the respective country over the 10-year period considered.

### Parameter fitting

There are 11 tuning parameters in the network model, 10 in the mean-field model, and 8 in the isolated models (Supplementary Table 1), fitted with simulated annealing running the full EKF pipeline and using the total forecasting error *E*(**T**) defined in (14) as the cost function (Supplementary Table 2). Since the different models have different numbers of fitted parameters, the data are divided into training and test data for a fair comparison. Data from the first four epidemic seasons (2014–2018) are used for parameter fitting, that is, **T** = {1, …, 202} in the cost function (until week 32 in 2018, see Fig. 3). The remaining six seasons (2018–2024), that is, **T** = {203, …, 499}, are used for evaluations. To ensure convergence, 15 simulated annealing runs with randomized starting points were run. Details of the simulated annealing are presented in Supplementary Note 4.

Note that the cost function *E*(**T**) for parameter fitting is based on the noise-free forecasts ${\widehat{y}}_{i}(t+\tau | t)$. If the covariance matrices *Q* and *U* and the initial uncertainty covariance *P*_0_ in the Kalman filter (see the algorithm in Supplementary Note 2) are multiplied by any positive constant, then the estimation error covariance *P*_*t*_, state prediction error covariance ${\widetilde{P}}_{t}$, and the output prediction error covariance *S*_*t*_ will be multiplied by the same constant at all times *t*. In the Kalman gain matrix ${\widetilde{P}}_{t}C(t){S}_{t}^{-1}$, however, the constant will cancel out due to the inverse of *S*_*t*_. As a consequence, the state estimates $\widehat{x}(t)$ and the noise-free forecasts ${\widehat{y}}_{i}(t+\tau | t)$ remain unchanged by such covariance scaling (see Supplementary Note 3 for details). It is therefore not possible to identify all covariance parameters at once by fitting the noise-free forecasts to data, but only their relative magnitudes. Without loss of generality, we therefore fix *σ*_*β*_ in *Q*_*i*_ in (8) to *Δtμ*^2^ to have a grounding value for the other covariance terms that are scaled by tuning parameters *κ*_1_ and *κ*_2_ in (8) and *ρ*_1_ and *ρ*_2_ in (10). Since the covariance terms can only be obtained up to constant multiplication by the parameter fitting procedure, a final noise calibration step will be carried out to find the proper (relative) scaling factor for the covariances.

### Noise calibration for probabilistic forecasts

The best way to produce probabilistic forecasts is to simulate trajectories from the full stochastic model and gather statistics from these simulations. However, as discussed above, the parameter fitting procedure only reveals the relative strengths of different noise components, but not the absolute noise level. For noise calibration, we therefore introduce a coefficient *K* used to multiply all noise terms in the model to control the uncertainty in the stochastic projections. As explained above, such noise scaling has no effect on the Kalman filter state estimate, since it does not affect the relative noise contributions. Hence, the parameters obtained by the fitting procedure can be used for probabilistic forecasts after the scaling. We simulate 1000 trajectories forward in time, initialized from a random initial state drawn from ${{{\mathcal{N}}}}\left(\widehat{x}(t),{K}^{2}{P}_{t}\right)$. Trajectories are then simulated forward in time using the stochastic model (6)–(7), where all noise terms *w*_*j*_ for *j* = 1, …, 4 and *w*_*β*_ are scaled with *K*. Both state-dependent and state-independent measurement noise corresponding to (10), again multiplied by *K*, is then added to the projected case numbers.

To find a good value for *K*, we generated 4-week ahead probabilistic forecasts on 58 different weeks covering the last two epidemic seasons (every week during high incidence and every second week during low incidence). Statistical coverage plots were then created by generating several different percentiles for the projections, and then calculating the frequency of occurrences where the real incidence (for forecast weeks 1–4) was above each percentile (Supplementary Fig. 5). This was repeated with several different values of *K*. Note that while the precise choice of *σ*_*β*_ does not influence the end results, having a reasonable value helps evaluate the sanity of the calibration results. With *σ*_*β*_ = *Δtμ*^2^, a change of one standard deviation in *β*_*i*_ in a time unit (a week) corresponds to the basic reproduction number *R*_0_ = *β*_*i*_/*μ* changing by 1. With such choice, *K* is expected to not deviate much from 1 (in orders of magnitude).

Due to nonlinear dynamics, the median of the 1000 trajectories does not coincide with the noise-free projection (*K* = 0) that was used for tuning the method. To account for potential bias, we also tried a scheme where all the simulated percentiles were adjusted by adding the difference of the noise-free projection and the median of the noisy simulations. From Supplementary Fig. 5, it can be observed that this adjustment indeed helps mitigate bias in the forecasts. Following the calibration result, *K* = 0.85 was selected as the reference value for the adjusted probabilistic forecasts.

### Reporting summary

Further information on research design is available in the Nature Portfolio Reporting Summary linked to this article.

## Results

### Including network effects improves forecasting performance

Similarly to earlier works such as Influcast^8^ and Respicast^9^, we evaluated forecasting performance using 1–4-week-ahead predictions for ILI across Europe. Forecasting performance was assessed from the adjusted average error between model predictions and actual data. The performance gain defined in (15) was quantified as the percentual reduction in error compared to a reference forecast, which uses the most recent observed incidence as the future prediction. This evaluation was conducted over 10 years of data, with separate analyses for training (first four epidemic seasons) and test datasets (remaining six epidemic seasons) to foster a fair comparison between different models with different numbers of fitted parameters, and for critical periods, namely wave onsets and epidemic peaks (as identified in Fig. 3).

**Fig. 4:**
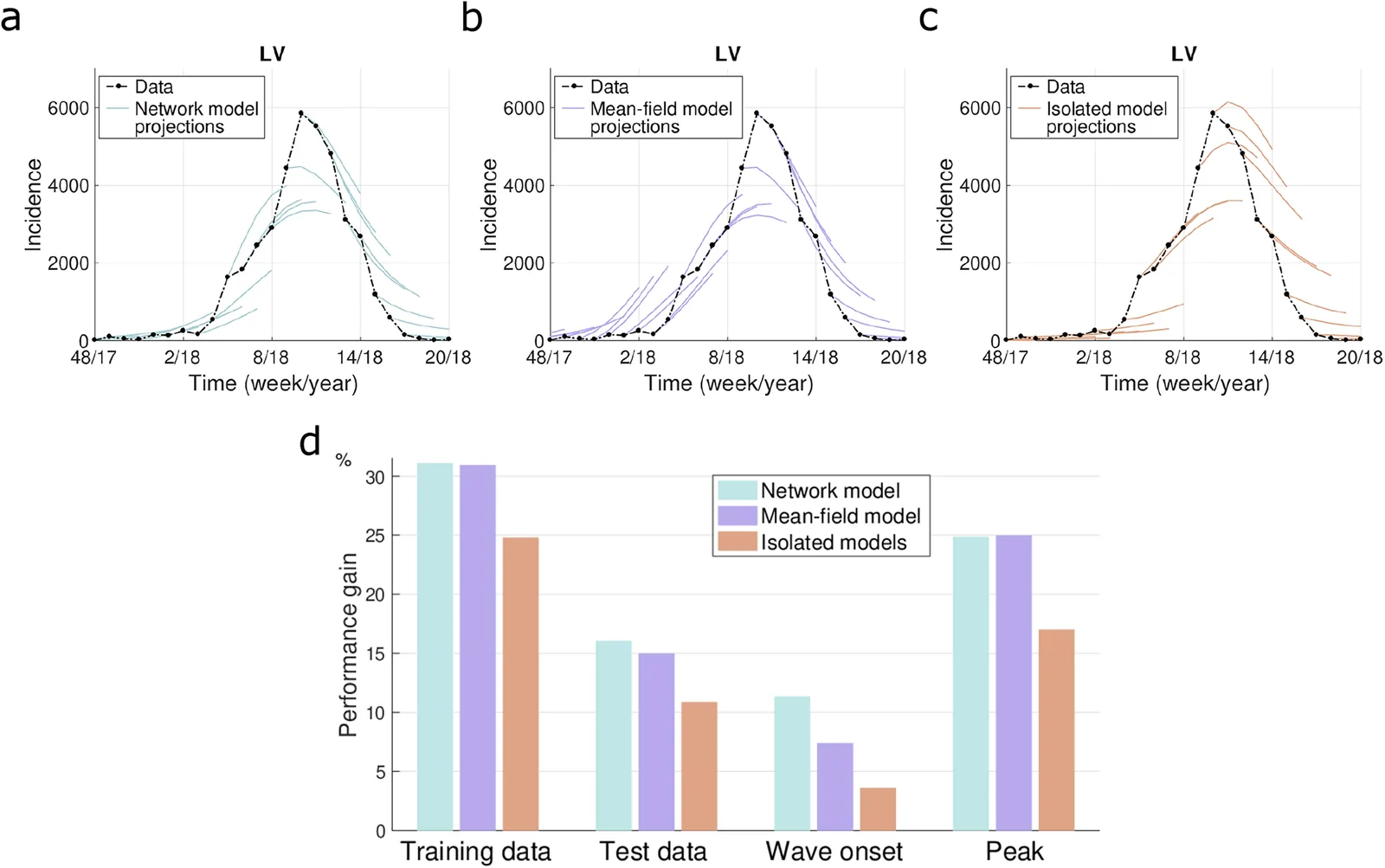
Forecasting results of the different models. **a**, **b**, **c** Comparison of 1–4-week-ahead forecasts by the network model, mean-field model, and isolated model, respectively for the 2017–2018 epidemic season in Latvia. The network model accurately captures the wave onset, outperforming the other models, which either underestimate or misalign the timing of the initial wave. In this epidemic season, the epidemic wave reached the Baltic states later than elsewhere in Europe (*cf*. Fig. 3), which explains the differences in the forecasts. Supplementary Fig. 4 shows the estimated *R*_0_(*t*) = *β*(*t*)/*μ* from different models for Latvia. **d** Performance gain of each model compared to the reference forecast, across training and test data (all countries), as well as during wave onset and peak periods. These results highlight the largely superior predictive accuracy of the network model and the mean-field model compared to to isolated models, particularly during critical epidemic phases.

The performance of each model—network model, mean-field model, and isolated models for 1–4-week-ahead forecasts—is shown in Fig. 4. The network and mean-field models consistently outperformed isolated models across all periods, highlighting the benefit of using trans-regional models across connected regions. The augmented models also prove useful to predict key moments of epidemic progression: peaks and onsets. Around epidemic peaks, the network and mean-field models achieved up to a 25% improvement in forecasting performance. Wave onset remains more difficult to forecast; nonetheless, in these periods, the network model provides the best results, clearly outperforming both the mean-field and isolated models. Knowing the mobility patterns among neighboring countries is thus particularly valuable for anticipating wave onsets.

**Fig. 5:**
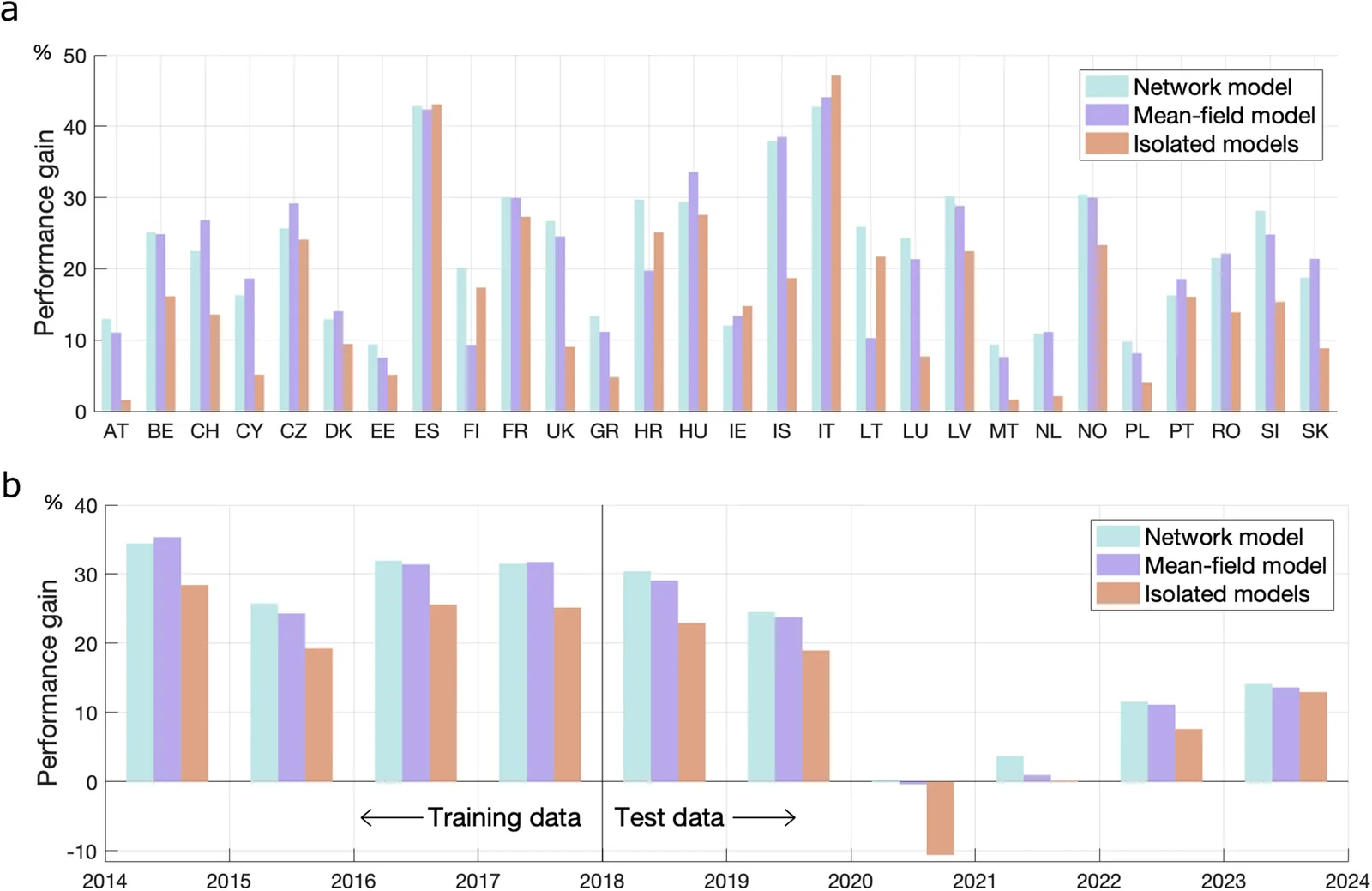
Detailed forecasting results. **a** Performance gain for each country over the entire 10-year period. **b** Season-by-season performance gains.

Performance gains varied by region and epidemic season (Fig. 5). The network model provided the highest gains in countries with strong cross-border interactions, such as Austria and Slovakia. In countries with a larger population size, performance tends to be slightly better overall, while the difference between the network model and isolated models is higher in countries with a smaller population size and with higher effect from the outside (see Supplementary Fig. 10). Predictions were more accurate during pre-pandemic seasons (2018–2019) compared to COVID-affected years, where reduced mobility and reporting inconsistencies impacted performance: since the mobility network is assumed to be static, it was less accurate when travel restrictions were in place. Also, the adaptive estimation scheme for the detection parameters *c*_*i*_(*t*) may be inadequate in extreme situations. Future improvements could incorporate a dynamic network evolution to reflect real-time changes in mobility patterns.

We also fitted model parameters using all 10 years of data, to check whether providing longer time series improved model predictions. The performance did not change considerably, and the same drop in the performance gain for test data period compared to training data period could still be observed. This confirms that the pandemic was the main cause of this performance drop, rather than potential overfitting. A similar performance was obtained even when only the first 2 years of data were used for parameter fitting. This is indicative of the parsimony of our model, and suggests that some country-specific parameters might be fitted based on forecasting performance without risk of overfitting. Results of these experiments are in Supplementary Fig. 7.

### Including network effects improves forecasting with missing data

Delays or missing values in reporting data are fairly common^9^. It is therefore crucial for an epidemic model to provide robust forecasts, even with missing data. Our network-based models leverage information from interconnected regions to impute missing data and provide accurate predictions, even when substantial portions of the dataset are unavailable. Here we study simplified missing data scenarios with randomly deleted data entries. While this setup does not exactly correspond to the observed reporting delays, it nevertheless indicates differences in performance for the different models in the presence of missing data. We simulated missing data scenarios by randomly deleting 20%, 40%, 60%, and 80% of the ILI data across all countries. Data that followed a period of at least 3 weeks of missing data were “protected” from deleting. Each scenario was repeated 20 times to account for variability. Forecasting performance was evaluated by the average forecasting error, obtained from the total error *E*(**T**) defined in (14) by normalizing by the number of summands, throughout the 10 years considered, **T** = {1, …, 499}. In addition, we measured how well the deleted data could be reconstructed using all available data until the reconstruction time. To enable direct comparison with forecasts, the same error metric is used, which is obtained by summing up (and normalizing by the number of summands) the reconstruction errors over all deleted data entries:

$${E}_{{{{\rm{r}}}}ec}={\sum }_{{{{\rm{Data}}}} \, (i,t) \, {{{\rm{deleted}}}}}\left|\sqrt{{y}_{i}(t)}-\sqrt{{\widehat{y}}_{i}(t| t)}\right|.$$

**Fig. 6:**
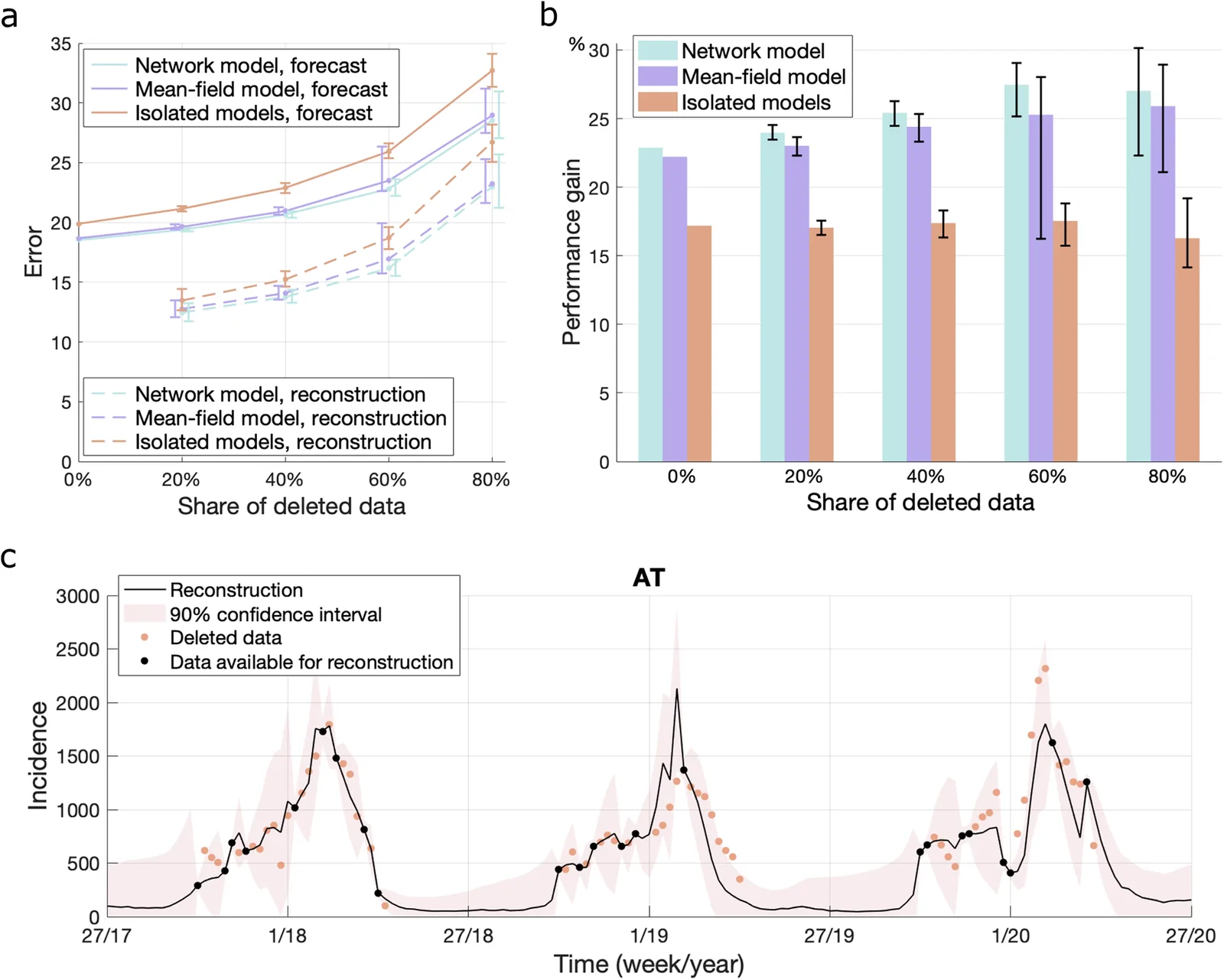
Performance of the models with different percentages of missing data. Confidence intervals (error bars) show the 90th percentiles over different randomized evaluations. **a** Average error for different percentages of deleted data; the network and mean-field models consistently outperform the isolated models, the error remaining relatively low even with 80% missing data. **b** Performance gain average with missing data compared to the reference forecast. **c** Example of data reconstruction for Austria over three epidemic seasons with 60% of the data deleted. The network model effectively reconstructs the missing data while maintaining confidence intervals. Observe the increasing width of the confidence interval in periods when data are missing.

As shown in Fig. 6a, the forecasting performance of the network-based model is robust to increasing percentages of missing data: prediction errors are rising only modestly. For example, with 40% data missing, the error increased by just 11.6%, compared to 15.1% for isolated models. The network model thus shows its ability to utilize inter-country dynamics to compensate for missing information. The reconstructed data closely aligned with true infection trends. Notably, reconstruction errors remained significantly lower than forecasting errors.

Moreover, the performance gain of the network model over the reference forecast increased with higher deletion levels, rising from 23 to 27.5% as data availability decreased from 100 to 40% (Fig. 6b), while the performance gain of the isolated models remained roughly constant at around 17%. The network model is also more consistent in its gain than the mean-field and isolated models, as appreciated by considering the variance in Fig. 6b. For reference, Fig. 6c shows an example for three epidemic seasons in Austria with 60% of the data deleted.

Our findings demonstrate the value of integrating information from neighboring countries, particularly in scenarios with incomplete datasets, and hence the utility of network-based models for improving forecasts and imputing missing data, which is particularly relevant for epidemic monitoring in regions with uneven surveillance or reporting practices.

### Probabilistic forecasts

Uncertainty quantification provided by the EKF allows the network-based model to provide valuable insight into the potential future trajectory of epidemics, accounting for inherent uncertainties and generating confidence intervals and probabilistic scenarios to guide decision-making.

**Fig. 7:**
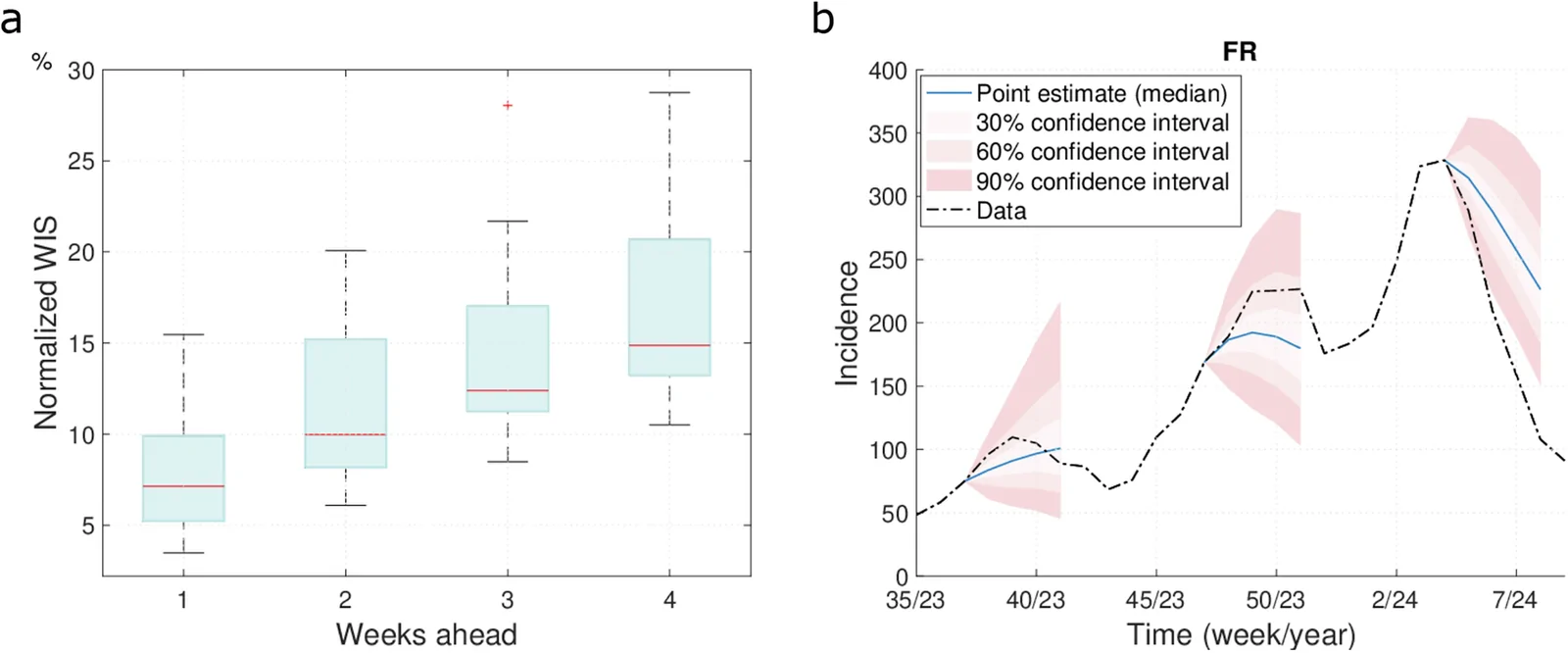
Evaluation and examples of probabilistic forecasts. **a** Average (over time) weighted interval scores (WIS) for 1–4-week ahead forecasts, normalized by average epidemic peak heights in each country. The boxplots show the median (over the 28 countries) with the red line, the 50th percentile with the box, and the minimum and maximum values with the whiskers, excluding outliers shown with red crosses. **b** Three 4-week-ahead probabilistic forecasts for France during the epidemic season 2023–2024.

Fig. 7a shows the normalized WIS for the 1–4-week ahead forecasts. The scores are first averaged over the entire 10-year period and then normalized by country with the average peak height over the 10 years. The statistics over all countries are shown by box plots. Supplementary Fig. 5 shows statistical coverage plots for the probabilistic forecasts, while Supplementary Fig. 6 shows separate box plots for each epidemic season. Fig. 7b shows examples of probabilistic forecasts for France during the 2023–2024 epidemic season. The model provides point estimates and different confidence intervals, capturing the uncertainty in the disease progression. The forecasts demonstrate the ability of the network model to anticipate epidemic trends, including wave peaks and durations, while offering actionable ranges for public health planning.

### Insights into model mechanisms and interpretability

Inspecting the results of parameter fitting in Supplementary Table 2, we notice that *k*_1_ is relatively big, strongly driving *β* towards the reference value (see (7)), in particular with the network models, where the reference value is heavily influenced by the network (*k*_2_ ≈ 0.8 in all network cases). Some parameters do not seem to be very well identifiable, resulting in large differences in the parameter values when the training is done using the first 4 years of data or all 10 years of data, while the performance did not change significantly. In particular, *γ*_2_ changes considerably in the network model, indicating that the inclusion of the neighbor network is not very important for forecasting performance.

Sensitivity analysis for the fitted parameters is provided in Supplementary Figs. 8 and 9 for the network model, also revealing a fairly weak sensitivity to changes in *γ*_2_. In addition, the results are not very sensitive to *κ*_2_ and *ρ*_2_ that regulate the state-independent noise terms in the covariances *Q*_*i*_ in (8) and *U*_*i*_ in (10). These terms were introduced to provide a lower bound for covariances during times of low incidence and thereby improve responsiveness at the onset of a new wave. In the isolated models, the values of these parameters are significantly higher, and indeed, setting *κ*_2_ = 0 makes the performance gain for the wave onset periods drop from 3.61 to 0.98% and setting *ρ*_2_ = 0 makes it drop to 1.48%. Setting both *κ*_2_ = *ρ*_2_ = 0 would make the EKF unstable for isolated models, since the measurement prediction error covariance *S*_*t*_ becomes at least nearly singular during times of low incidence (see the Algorithm in the Supplementary Note 2).

The mechanistic design of the model allows for a detailed inspection of the factors driving its performance. To better understand these dynamics, we conducted additional experiments, whose results are shown in Supplementary Fig. 7. Firstly, the network model improves performance more for countries where the outside effect is larger (Supplementary Fig. 10c). To investigate whether this result is merely spurious, we ran the method after scaling the network matrices *G*^(1)^ + *G*^(2)^, and *G*^(3)^ row-wise to a fixed percentage of the populations of the respective countries. The forecasting performance decreased slightly compared to the network model, suggesting that the network model accurately captures the true effect of mobility. Secondly, the inclusion of network effects in the dynamics of the transmission parameter *β* was found to play a crucial role in enhancing forecasting capabilities. When this effect was removed by setting *k*_2_ = 0 in (7), the model’s performance dropped markedly. Finally, we tested a hybrid model where the mean-field network informed only the *β*-parameter dynamics, while the *S* → *I* dynamics were governed by isolated models. The performance of this model was mostly comparable to the full network model. This observation suggests that including network effects into the *β*-parameter dynamics has a stronger impact than including the mobility component into the *S* → *I* dynamics. This is also supported by a fairly small sensitivity to *γ*_1_ and *γ*_2_ parameters compared to the sensitivity to the *k*_*j*_ parameters in the *β*-dynamics. Note that *γ*_*j*_-parameter magnitudes are normalized out in the *β*-dynamics (7), and therefore the sensitivity to *γ*_*j*_-parameters does not reflect the relevance of the network component in (7).

Interestingly, the detailed network model outperformed all alternatives during wave onset periods, highlighting its strength in capturing the early dynamics of epidemic waves.

## Discussion

This work shows the advantages of a networked metapopulation SIRS model, integrated with an EKF, for forecasting different aspects of the dynamics of ILI across Europe. The model leverages empirical data on mobility and disease incidence to incorporate both local dynamics and cross-border interactions and thus improve forecasting accuracy. Comparative analyses show that the network-based model provides a mild advantage in forecasting epidemic progression, compared to mean-field models, but outperforms isolated and mean-field models during critical periods such as wave onsets and epidemic peaks. Additionally, the network model is more robust to missing data and provides reliable forecasts even with substantial data unavailability.

Our findings highlight that integrating data from several connected regions into network-based epidemic models improves accuracy and interpretability. In a probabilistic forecasting framework, the EKF enables the model to adapt dynamically to new data, providing real-time forecasts with quantified uncertainty that is critical for proactive responses. Our proposed framework is highly relevant for epidemic monitoring and regional and global public health decision-making, with several applications in forecasting. By providing a spectrum of plausible epidemic trajectories, it supports scenario analysis and planning, including: (1) Early warnings: identifying potential wave onsets and peak periods with quantified uncertainty; (2) Resource allocation: informing healthcare capacity planning by projecting worst-case and best-case scenarios; (3) Scenario modeling: evaluating the impact of interventions, such as travel restrictions or vaccination campaigns, by simulating alternative futures.

The demonstrated benefits of network-based approaches are expected to influence the design of future epidemic forecasting systems, particularly in interconnected regions. Our results encourage further investigation and the adoption of similar frameworks for diseases such as COVID-19, dengue, and seasonal influenza (after appropriately incorporating adequate models into the Kalman filter pipeline). The reconstructed European mobility network can also serve as a foundation for further studies, fostering collaboration and data sharing among public health agencies.

Static network structures limit the accuracy of predictions during periods of rapid mobility changes, such as those caused by pandemics or natural disasters. Incorporating dynamic network adjustments, informed by real-time mobility data, could enhance model adaptability and performance. Our pipeline also implicitly assumes that data collection is consistent between epidemic seasons. The adaptive scheme for estimating the detection parameters *c*_*i*_(*t*) may not be able to cope with drastic changes that are sometimes observed in the data. Any knowledge of changes can be manually incorporated to improve performance. Also, we did not fit parameters that are specific to countries or population demographics, based on forecasting performance. According to the country-specific parameter sensitivity analysis (Supplementary Fig. 9), doing so may lead to additional improvement.

Moreover, the model currently assumes homogeneous mixing within populations, which may oversimplify complex interactions, particularly in urban areas. It can be observed (Fig. 5a and Supplementary Fig. 10) that the performance is on average slightly better for countries with larger population than with smaller population (although the difference is mainly driven by the good performance for Italy and Spain), but the performance improvement of the network model compared to isolated models is generally smaller for larger populations. In countries with larger population, the data are averaged over larger numbers, and are typically more regular than in countries with smaller population. While regular data improve forecasting performance in general (like for Italy and Spain), there may be large regional differences within bigger countries, and more targeted forecasts could be more useful. Including network effects tends to improve the performance more significantly when the population size is small because the relative proportion of external (vs. internal) contributions to contagion (which depends on the amount of daily travelers across the borders, including cross-border workers) is likely to be larger in geographically small countries, which typically have a smaller population size; hence, the outcome does not simply depend on the population size, but on multi-pronged factors that implicitly account also for geographical size and intensity of exchanges with neighboring regions. While it is likely that the effect of mobility would be stronger with finer spatial granularity, developing interconnected models for given regions and studying the effect of spatial granularity are two distinct issues, and also the latter would be worth exploring in the future. Future work could integrate finer-grained data, such as inter-city mobility patterns, or other complementary data such as holiday schedules like in ref. ^40^, or humidity data like in refs. ^5,29,34,40^. Additionally, the absence of certain countries from the network, due to data unavailability, may introduce regional biases. Expanding public databases and standardizing data collection practices across Europe would address this issue.

Lastly, while the model performs well for ILI, its applicability to other diseases with different transmission dynamics, such as vector-borne or chronic diseases, remains to be explored. Adjusting the framework to accommodate diverse epidemiological characteristics would broaden its impact.

## Supplementary information

Transparent Peer Review file

Supplementary information

Description of Additional Supplementary files

Supplementary Data 1

Reporting summary

## Data Availability

The 10 years of ILI incidence data (detected cases over time, with an average weekly reporting frequency) for 31 regions used for the results in this article originate from the Respicast platform^10^. To construct the network models, long-range mobility traffic data (number of flights and ferry travels) are publicly accessible from refs. ^43–45^; cross-border commuting information (flows over time) is publicly accessible at^46,47^; population densities of NUTS 3 regions are publicly available from refs. ^48,49^. A copy of the incidence data and the processed network components are provided along with the code. A separate figure source file is available online as Supplementary Data.

## Code availability

All code required to reproduce the results presented here is available at gitlab.com/uniluxembourg/lcsb/systems-control/epinetekf together with a user guide^57^.
