# Publisher Correction: Networked SIRS model with Kalman filter state estimation for epidemic monitoring in Europe

**DOI:** 10.1038/s43856-026-01893-z

**Published:** 2026-09-07

**Authors:** Atte Aalto, Daniele Proverbio, Giulia Giordano, Alexander Skupin, Jorge Gonçalves

**Affiliations:** 1 Luxembourg Centre for Systems Biomedicine, University of Luxembourg, Belvaux, Luxembourg; 2 Department of Industrial Engineering, University of Trento, Trento, Italy; 3 Department of Physics and Material Sciences, University of Luxembourg, Belvaux, Luxembourg; 4 Department of Neurosciences, University of California, San Diego, CA USA; 5 Department of Plant Sciences, University of Cambridge, Cambridge, UK

**Keywords:** Epidemiology, Viral infection

Correction to: *Communications Medicine* https://doi.org/10.1038/s43856-026-01611-9, published online 08 May 2026.

In the PDF version of the article, a portion of the text was inadvertently hidden. The article has now been updated.

