# Supplementary material for: Networked SIRS model with Kalman filter state estimation for epidemic monitoring in Europe
Source: Commun Med (Lond). 2026 May 8;6:398. doi: 10.1038/s43856-026-01611-9 (PMC13376820; doi:10.1038/s43856-026-01611-9)
Supplement: Supplementary file 2 — Supplementary information [file 43856_2026_1611_MOESM2_ESM.pdf]

Supplementary material for:

Networked SIRS model with Kalman filter state estimation for epidemic monitoring in Europe

Atte Aalto[1,*], Daniele Proverbio[2], Giulia Giordano[2], Alexander Skupin[1,3,4], and Jorge Gonçalves[1,5]

1: Luxembourg Centre for Systems Biomedicine, University of Luxembourg, Belvaux, Luxembourg
2: Department of Industrial Engineering, University of Trento, Trento, Italy
3: Department of Physics and Material Sciences, University of Luxembourg, Belvaux, Luxembourg
4: Department of Neurosciences, University of California, San Diego, USA
5: Department of Plant Sciences, University of Cambridge, Cambridge, UK

*Corresponding author: atte.aalto@uni.lu

Supplementary Note 1: Handling outliers in the neighbor network construction

On the Italian–Slovenian border, the Trieste province (NUTS3 code ITH44) is very densely populated and narrow, being only a couple of kilometers wide. Its neighboring province is Gorizia (ITH43), whose size is relatively small as well. Flows between these regions and Slovenia are thus outliers in our method. Hence, both Trieste province and Gorizia province were joined with the larger Udine province (ITH42) to obtain reasonable flow estimates. Similarly, the Geneve canton (CH013) on Switzerland's border with France was joined with the neighboring Vaud canton (CH011), and the canton of Basel–Stadt (the city of Basel, CH031) was joined with the surrounding canton of Basel–Land (CH032). On the French–Belgian border, the densely populated and narrow department of Nord (FRE11) was joined with the neighboring department Pas-de-Calais (FRE12), even though the latter does not share a border with Belgium.

The Zuid–Limburg region (around Maastricht) in the Netherlands forms a protrusion into Belgium, and has a very high population density. To account for the protruding shape, the length of the border between the Zuid–Limburg region and Belgium was reduced by a third. The border length between the Torino province (ITC11) and France was reduced from 141 km to 111 km, excluding some excessive turns the border makes. Similarly, the border length between the Belgian arrondissement of Antwerp and the Netherlands was reduced from 51 km to 32 km, excluding some turns.

Supplementary Note 2: Algorithm

Set $P_0 \in \mathbb{R}^{84\times 84}$ and $\hat{x}(0)$;
while $t \leq T_{\max}$ **do**
  set $\tilde{x} = \hat{x}(t-1)$;
  set $\tilde{P}_t = P_{t-1}$;
  for *i=1,...,7* **do**
    Prediction error covariance: $\tilde{P}_t = J_f(\tilde{x})\tilde{P}_t J_f(\tilde{x})^\top + Q(\tilde{x})$;
    Prediction for time $t-1+i/7$: $\tilde{x} = f(\tilde{x})$;
  end
  Measurement prediction error covariance: $S_t = C(t)\tilde{P}C(t)^\top + U(t)$;
  State update: $\hat{x}(t) = \tilde{x} + \tilde{P}_t C(t)^\top S_t^{-1}\big(y(t) - C(t)\tilde{x}\big)$;
  Error covariance update: $P_t = \tilde{P}_t - \tilde{P}_t C(t)^\top S_t^{-1} C(t)\tilde{P}_t$;
  if $S_i \geq 0.4 N_i$ *for all* i **then**
    $t = t+1$;
  else
    Update $c_i(t)$ upward;
    $t = t-12$;
  end
  if $t \in \{30, 82, 134, 186, 238, 290, 342, 394, 446, 498\}$ **then**
    Update $c_i(t)$ downward if necessary;
  end
end

Algorithm 1: The Extended Kalman filter for the networked SIRS model. $J_f(x)$ is the Jacobian of the function f, evaluated at x. The algorithm is standard, but the prediction step consists in solving the SIRS model one week forward in time between measurement times. The weeks for a downward update of $c_i(t)$ are pre-determined. They are in the middle of summer during a low-incidence period.

Supplementary Note 3: Note on the redundancy of noise scaling

Consider the Extended Kalman filter error covariance equations:

$$\begin{cases} \tilde{P}_1(t) = J_f(\hat{x}_1(t-1))P_1(t-1)J_f(\hat{x}_1(t-1))^\top + Q \\ P_1(t) = \tilde{P}_1(t) - \tilde{P}_1(t)C^\top\big(C\tilde{P}_1(t)C^\top + U\big)^{-1}C\tilde{P}_1(t) \end{cases} \tag{1}$$

where $J_f(\hat{x}_1(t-1))$ is the Jacobian of the dynamics function, evaluated at the current state estimate $\hat{x}_1(t-1)$. Consider then equations where the covariance terms Q and U are scaled by an arbitrary constant $K^2 > 0$:

$$\begin{cases} \tilde{P}_2(t) = J_f(\hat{x}_2(t-1))P_2(t-1)J_f(\hat{x}_2(t-1))^\top + K^2Q \\ P_2(t) = \tilde{P}_2(t) - \tilde{P}_2(t)C^\top\big(C\tilde{P}_2(t)C^\top + K^2U\big)^{-1}C\tilde{P}_2(t) \end{cases} \tag{2}$$

We present an inductive proof on the equivalence of the Kalman filter state estimates $\hat{x}_1(t)$ and $\hat{x}_2(t)$ corresponding to the two filters. So assume that the state estimates coincide for time $t-1$, that is, $\hat{x}_1(t-1) = \hat{x}_2(t-1)$, in which case the Jacobian matrices in (1) and (2) are the same. Assume also that $P_2(t-1) = K^2P_1(t-1)$.

Now, from the first equation in (2), we have

$$\begin{aligned} \tilde{P}_2(t) &= J_f(\hat{x}_2(t-1))P_2(t-1)J_f(\hat{x}_2(t-1))^\top + K^2Q \\ &= K^2J_f(\hat{x}_1(t-1))P_1(t-1)J_f(\hat{x}_1(t-1))^\top + K^2Q \\ &= K^2\tilde{P}_1(t) \end{aligned}$$

and from the second equation in (2):

$$\begin{aligned} P_2(t) &= \tilde{P}_2(t) - \tilde{P}_2(t)C^\top\big(C\tilde{P}_2(t)C^\top + K^2U\big)^{-1}C\tilde{P}_2(t) \\ &= K^2\tilde{P}_1(t) - K^2\tilde{P}_1(t)C^\top\big(K^2C\tilde{P}_1(t)C^\top + K^2U\big)^{-1}K^2C\tilde{P}_1(t) \\ &= K^2\Big(\tilde{P}_1(t) - \tilde{P}_1(t)C^\top\big(C\tilde{P}_1(t)C^\top + U\big)^{-1}C\tilde{P}_1(t)\Big) \\ &= K^2P_1(t). \end{aligned}$$

The Kalman gain for the second filter at time t is

$$\begin{aligned} \tilde{P}_2(t)C^\top S_2(t)^{-1} &= \tilde{P}_2(t)C^\top\big(C\tilde{P}_2(t)C^\top + K^2U\big)^{-1} \\ &= K^2\tilde{P}_1(t)C^\top\big(K^2C\tilde{P}_1(t)C^\top + K^2U\big)^{-1} \\ &= \tilde{P}_1(t)C^\top\big(C\tilde{P}_1(t)C^\top + U\big)^{-1} \end{aligned}$$

which is precisely the Kalman gain for the first filter at time t, and thus the state estimates coincide also for time t. We therefore conclude inductively that the state estimates coincide for all times, given that the initial covariances satisfy $P_2(0) = K^2P_1(0)$ and the state estimates are initialized from the same state, $\hat{x}_1(0) = \hat{x}_2(0)$.

Supplementary Note 4: Simulated annealing for parameter fitting

The 8, 10 or 11 tuning parameters (depending on the model) are fitted by simulated annealing. To this end, we define a cost function $J(\bar{p})$ consisting of the sum of total forecasting errors over all 28 countries, $J(\bar{p}) = \sum_{i=1}^{28} E_i(\bar{p})$ where E_i is defined in Eq. (14) in the main text and $\bar{p} \in \mathbb{R}^{11}$ is the tuning parameter vector. That is, the full EKF pipeline is run with parameters $\bar{p}$, and the forecasting errors are collected and summed up. Simulated annealing is used for optimization because the cost function is not smooth (not even continuous) due to the triggering mechanism used for updating the $c_i(t)$ parameters.

Simulated annealing is closely related to MCMC sampling. The idea is to draw samples from a distribution $\exp\big(-J(\bar{p})/T\big)$ where T is temperature. As the algorithm proceeds, the temperature is slowly decreased, whereby the sample chain converges to a local optimum as $T \to 0$. By initializing the sampling from the optimal point from several randomly drawn candidates and by restarting the chain several times, we help the algorithm in exploring the parameter space comprehensively.

The initial parameter vector is selected by drawing 60 candidate parameter vectors $\bar{p}_j$ where each parameter p is randomized by $p = 2^{-a+2ar} p_{\text{ref}}$, where $r \sim U(0,1)$. The parameter minimizing $J(\bar{p}_j)$ is taken as the initial parameter vector for the simulated annealing step. In total, 15 simulated annealing runs are done sequentially, where on the first five runs, p_{ref} is an initial reference value, and on later runs, the optimal parameter vector found so far was used as p_{ref}. The variability of the initial parameter was controlled by the box width parameter a which was 1 on the first seven runs, 0.5 on the next four runs, and 0.25 on the last four runs. Once the initial parameter was chosen, the simulated annealing proceeds as shown in the algorithm below. There $D \in \mathbb{R}^{11\times 11}$ is a diagonal matrix containing step size scales for each parameter, which are then modulated by a factor s. This factor is adaptively adjusted during the simulated annealing run based on the moving average of the acceptance rate.

```
Set p̄_old to the initial value;
Set step size s = 1;
for j = 1, ..., 350 do
    Draw new candidate p̄_new = p̄_old + sDr, where r ~ N(0, I);
    Calculate J(p̄_new);
    if exp( (J(p̄_old) − J(p̄_new)) / T ) > v where v ~ U(0,1) then
        Set p̄_old = p̄_new;
        Set T = 0.98T;    (decrease temperature)
        a_j = 1;    (acceptance indicator)
    else
        a_j = 0;
    end
    if Σ_{k=j−9}^{j} a_k ≤ 1 then
        Set s = 0.75s;    (decrease step)
    end
    if Σ_{k=j−9}^{j} a_k ≥ 7 then
        Set s = 1.1s;    (increase step)
    end
end
```

Algorithm 2: Simulated annealing for parameter fitting.

Supplementary Table 1: Summary of the 12 tuning parameters fitted by simulated annealing. Equation references refer to the main text.

Group	Symbol	Explanation	Ref. Eq.
Network	γ_1	Coefficient for the travel and cross-border work networks	(1)
	γ_2	Coefficient for the neighbour network	(1)
	γ	Coefficient for the mean-field network	(2)
State noise	κ_1	Coefficient for the Langevin covariance	(6)
	κ_2	Coefficient for the state-independent noise covariance	(6)
Measurement noise	ρ_1	Coefficient for the state-dependent measurement noise	(10)
	ρ_2	Coefficient for the state-independent measurement noise	(10)
β-dynamics	k_1	Coefficient for β-dynamics	(7)
	k_2	Coefficient for neighbour average vs. grounding value in β-dynamics	(7)
	k_3	Coefficient for grounding value of β	(7)
Adaptive $c_i(t)$ estimation	α_1	Coefficient for initial value for $c_i(t)$	(11)
	α_2	Transformation exponent between case numbers and $c_i(t)$	(12),(13)

Supplementary Table 2: Fixed and fitted parameter values for different models. The fixed parameters are the same for all models. Note that with the two mean-field models, the first parameter is γ, and it is not comparable with values of γ_1. Also the γ_j values for the scaled network are not comparable with other network models. When the network is only used in β-dynamics, γ is set to 1, since its precise value would be lost in the normalization in Eq. (7) in the main text.

Fixed parameters

$\mu = 0.06$	$\varphi = 0.0116$	$\sigma_\beta = 5.143 \cdot 10^{-4}$	$\alpha_3 = 0.756^{\alpha_2}$	$\Delta t = 1/7$

Fitted parameters

Model	γ / γ_1	γ_2	κ_1	κ_2	ρ_1	ρ_2	k_1	k_2	k_3	α_1	α_2
Factor	1	1	10^4	10^{-7}	10^{-2}	10^{-5}	10^{-1}	1	1	1	1
Network	1.787	0.666	1.580	0.722	8.32	2.85	1.71	0.832	1.325	0.812	1.442
Isolated	0	0	3.854	55.74	46.52	19.90	0.33	0	1.393	0.670	0.794
Mean-field	6.246	0	3.218	14.26	8.93	7.25	0.90	0.766	1.194	0.809	1.568
Scaled network	2.943	0.570	2.447	1.41	26.3	5.72	1.15	0.801	1.260	0.817	1.514
Case $k_2 = 0$	13.36	3.605	5.763	6.23	73.48	25.19	0.198	0	1.086	0.641	0.975
Mean-field in β	1	0	1.530	34.89	5.22	4.46	1.10	0.858	1.433	0.642	1.038
Network, full data	1.754	0.064	1.965	0.297	6.64	5.25	2.17	0.846	1.312	0.884	0.944
Network, half data	1.508	0.158	1.053	55.08	0.162	3.03	2.86	0.885	1.370	0.808	1.099
Isolated, full data	0	0	2.745	43.48	34.64	18.38	0.443	0	1.398	0.807	0.913
Isolated, half data	0	0	4.800	117.7	48.42	22.44	0.427	0	1.386	0.771	0.898

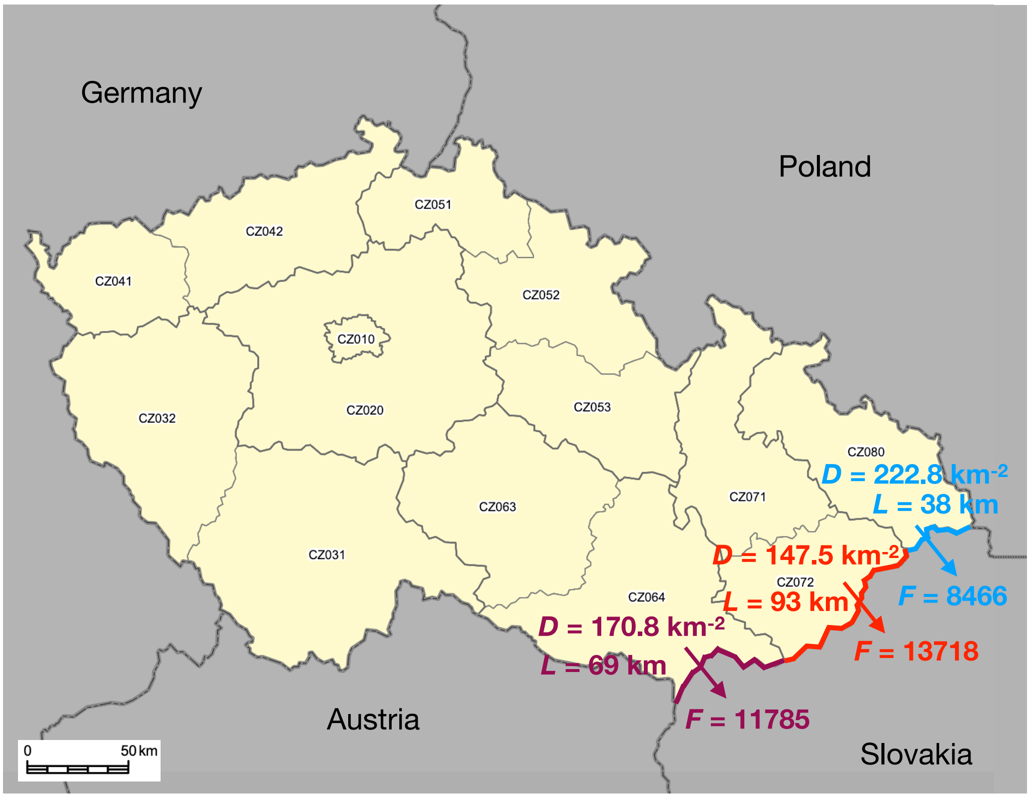

Supplementary Figure 1: NUTS 3 classification of regions for Czechia, and an illustration on the construction of the neighbor graph for the Czechia–Slovakia border. The flux across the border (F) is obtained as the product of the region's population density (D) and the length of the border segment between the region and the neighboring country (L). The map is from NUTS (https://ec.europa.eu/eurostat/web/nuts ▹ Maps), with our own additions.

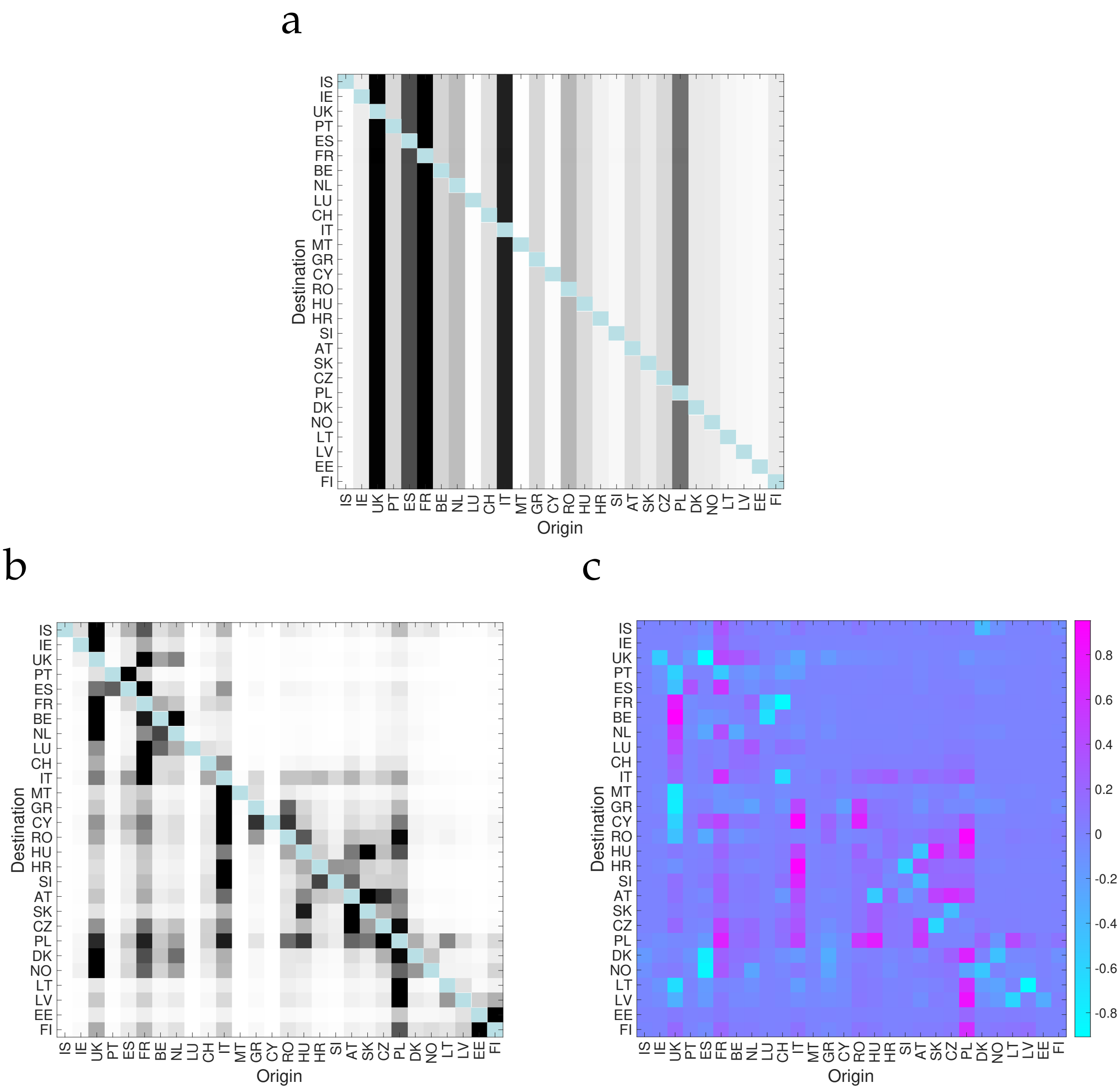

Supplementary Figure 2: **a:** Heatmap for the mean-field network matrix. Due to the scaling of each row to have maximum value one, each column depends only on the population of the country of origin. **b:** Matrix heatmap for the gravity network, that would offer an alternative for our travel-based network. Each network entry (i, j) is given by $N_j N_i / d_{i,j}^{\rho}$ where $d_{i,j}$ is the distance between countries i and j and ρ is a tuning parameter ($\rho = 1$ is used for this visualization, in line with Xia et al. (2004) (see the reference in the main text)). The entries are then normalized by row. **c:** Difference of the normalized gravity network and travel-based network. Notably, the gravity model underestimates the connection from UK to many south-European destinations like Malta, Greece, Cyprus, and Portugal while overestimating the connection from UK to its nearest neighbors, France, Belgium, and Netherlands. The connections from Italy and Poland are generally overestimated by the gravity model.

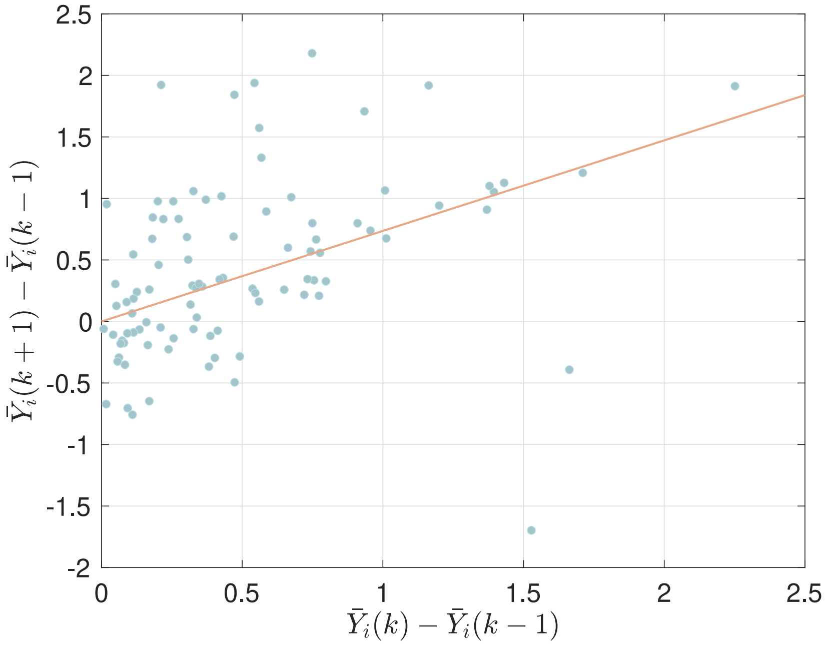

Supplementary Figure 3: Parameter α_3 was estimated directly from data by calculating the correlations between changes over two years and (positive) changes over one year. That is, yearly total numbers of cases were calculated for each country and denoted by $\bar{Y}_i(k) = \sum_{t=52(k-1)}^{52k} y_i(t)$, where $k = 1, ..., 10$ denotes the year. We then formed pairs $\big(\bar{Y}_i(k) - \bar{Y}_i(k-1), \bar{Y}_i(k+1) - \bar{Y}_i(k-1)\big)$ for each country for $k = 2, ..., 9$, and then calculated the regression line for these points including only those for which $\bar{Y}_i(k) - \bar{Y}_i(k-1) > 0$. The regression coefficient 0.756 (raised to power α_2) is used as the value for the parameter α_3.

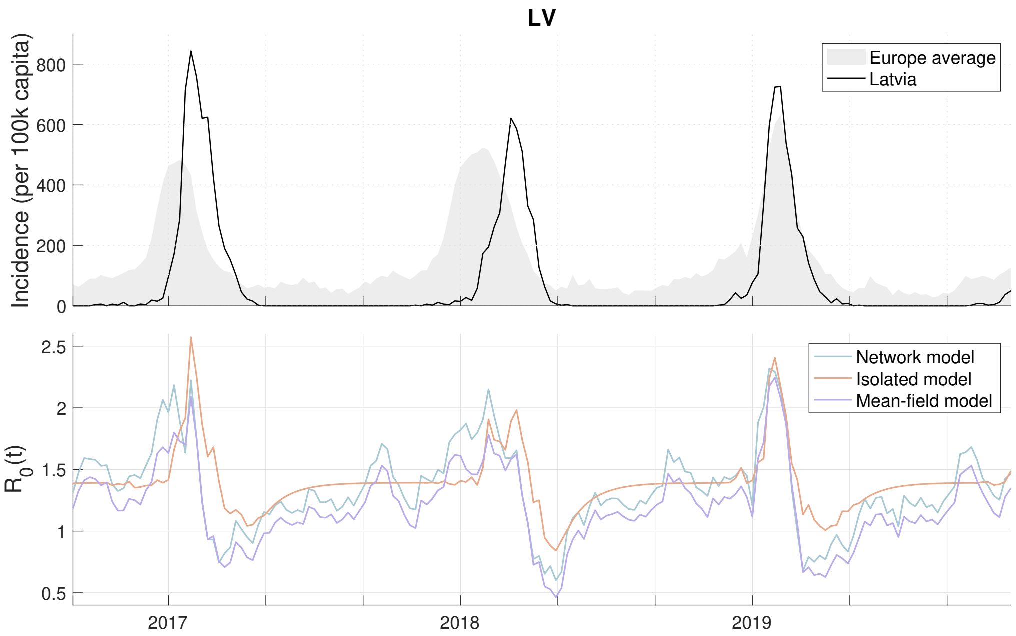

Supplementary Figure 4: Top panel shows the incidence data for Latvia together with the European average. The bottom panel shows the estimated $R_0(t) = \beta(t)/\mu$ for the different models. On the two epidemic seasons 2016–2018, the epidemic wave arrives rather late in Latvia, allowing the two network models to anticipate better. During the season 2018–2019, the wave coincides with the European average, and all $R_0(t)$-estimates are very similar. Projections for the 2017–2018 season are shown in Fig. 4 of the main text.

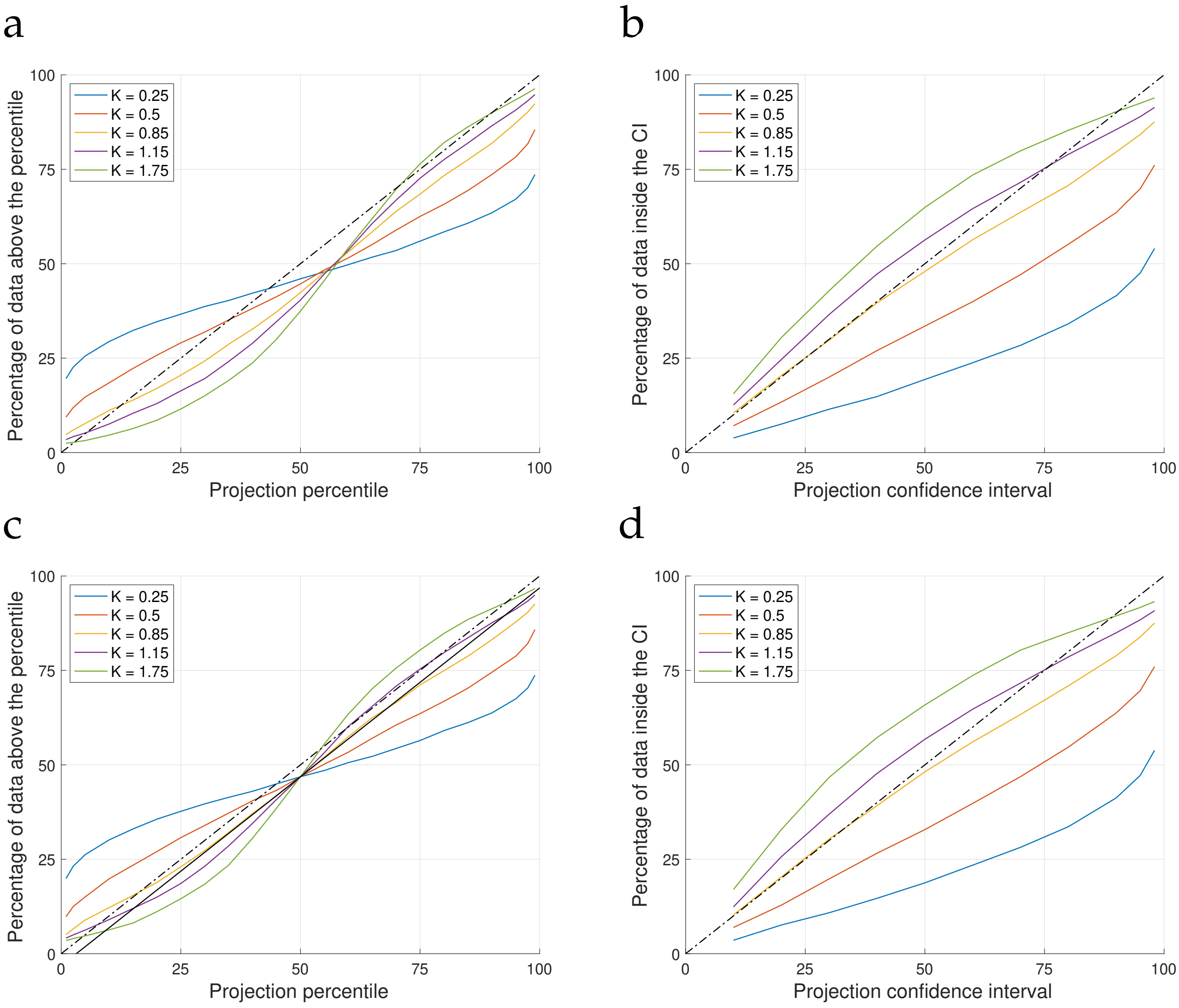

Supplementary Figure 5: Calibration results shown in coverage plots. In all plots, the black dash-dotted line indicates a perfectly calibrated (and unbiased in panels a and c) forecast. **a:** Share of cases when the true incidence is above different forecast percentiles for the non-adjusted forecasts. With different tested values of K, the true incidence is above the forecast median (Projection percentile 50) in 37.5–46.0% of forecasts, indicating some bias (underestimation) in the forecasts. **b:** Share of cases when the true incidence is inside different confidence intervals for the non-adjusted forecasts. **c:** Share of cases when the true incidence is above different forecast percentiles for the adjusted forecasts. Due to the adjustment, all curves coincide at the median, and 46.8% of true incidence is above the forecast median, which is a clear improvement on the non-adjusted forecasts, in particular for appropriate values of K. The black solid line is parallel to the line corresponding to perfect calibration, but it is offset to pass through the forecast median point. The case with $K = 0.85$ matches this line at best (also $K = 0.7$, $K = 1$, $K = 1.3$, and $K = 1.5$ were tested but are not shown). **d:** Share of cases when the true incidence is inside different confidence intervals for the adjusted forecasts.

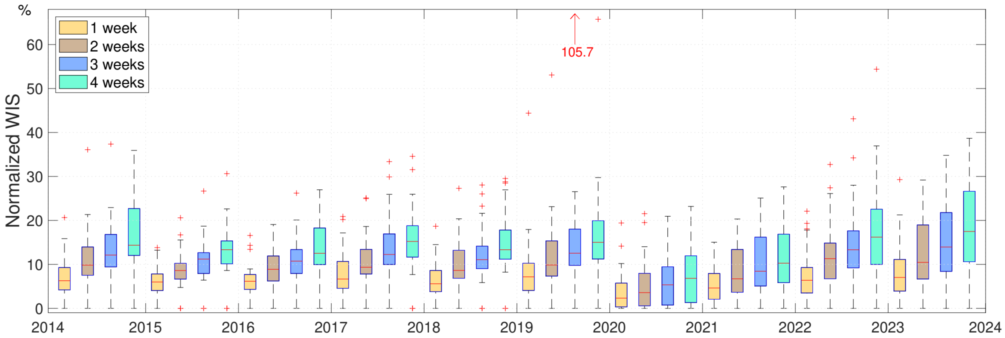

Supplementary Figure 6: The average weighted interval scores for each epidemic season normalized, for each country, by the average amplitude of the epidemic waves. The scores are lower for the two COVID-affected seasons, which is due to the smaller amplitude of the epidemic waves during these years. The largest outliers correspond to Slovenia in the 2019–2020 season and for Austria in the 2022–2023 season. These are due to exceptionally high epidemic waves in these countries compared to other years, which gave rise to higher WIS compared to the average wave amplitude used for normalization.

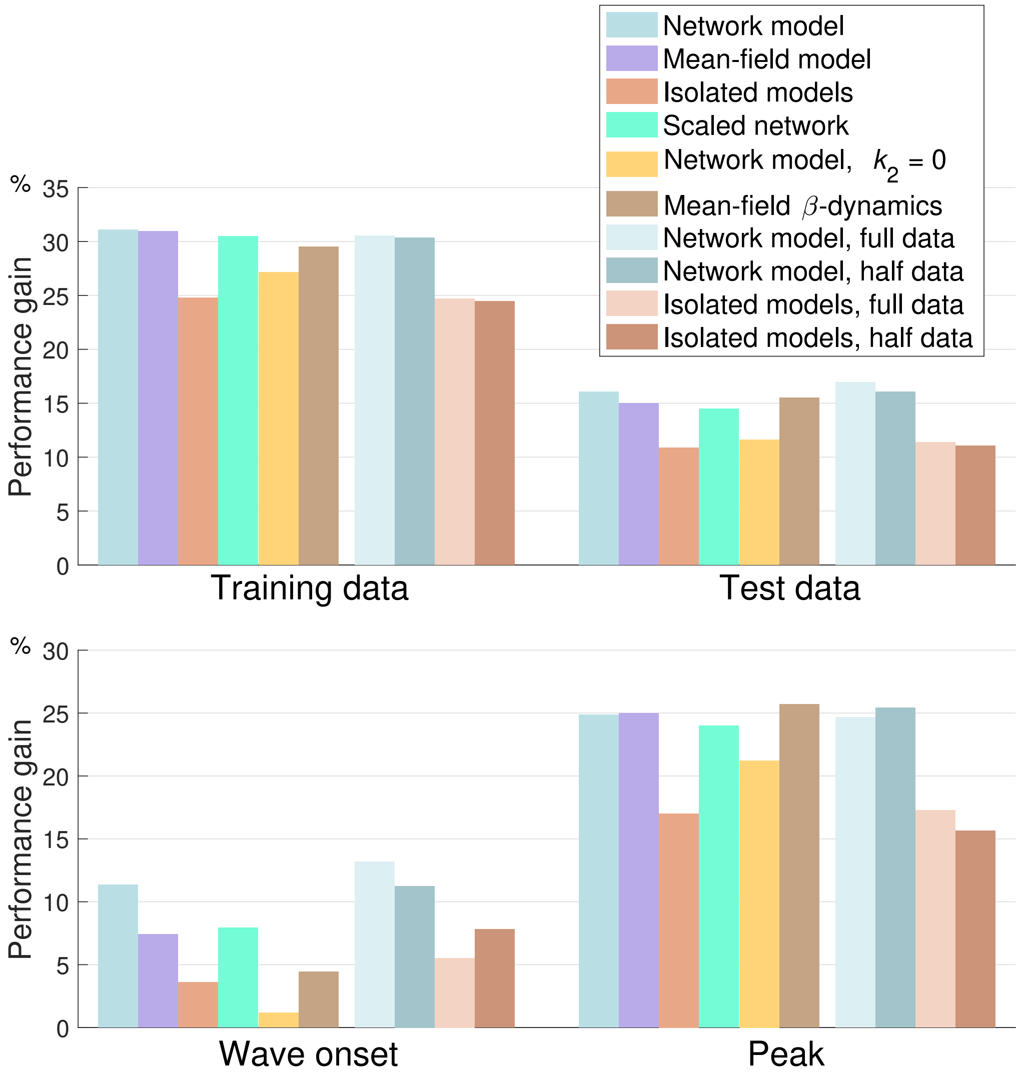

Supplementary Figure 7: Performance for the supplementary experiments for interpretability. The first three bars correspond to the results shown in Fig. 4 of the main text. The scaled network is obtained by scaling the rows of $G^{(1)} + G^{(2)}$ and $G^{(3)}$ to a fixed percentage of the populations of the respective countries. Setting $k_2 = 0$ removes the effect of the network in the β-parameter dynamics. The "Mean-field β-dynamics" refers to a model where the mean-field network is used, but only in β-parameter dynamics, while the calculation of $F_{S_i \to I_i}$ is done as with isolated models. The last four bars are obtained by using either all ten years of data for parameter tuning, or only the first two years. The evaluations shown here are still done using the original split between training and testing sets.

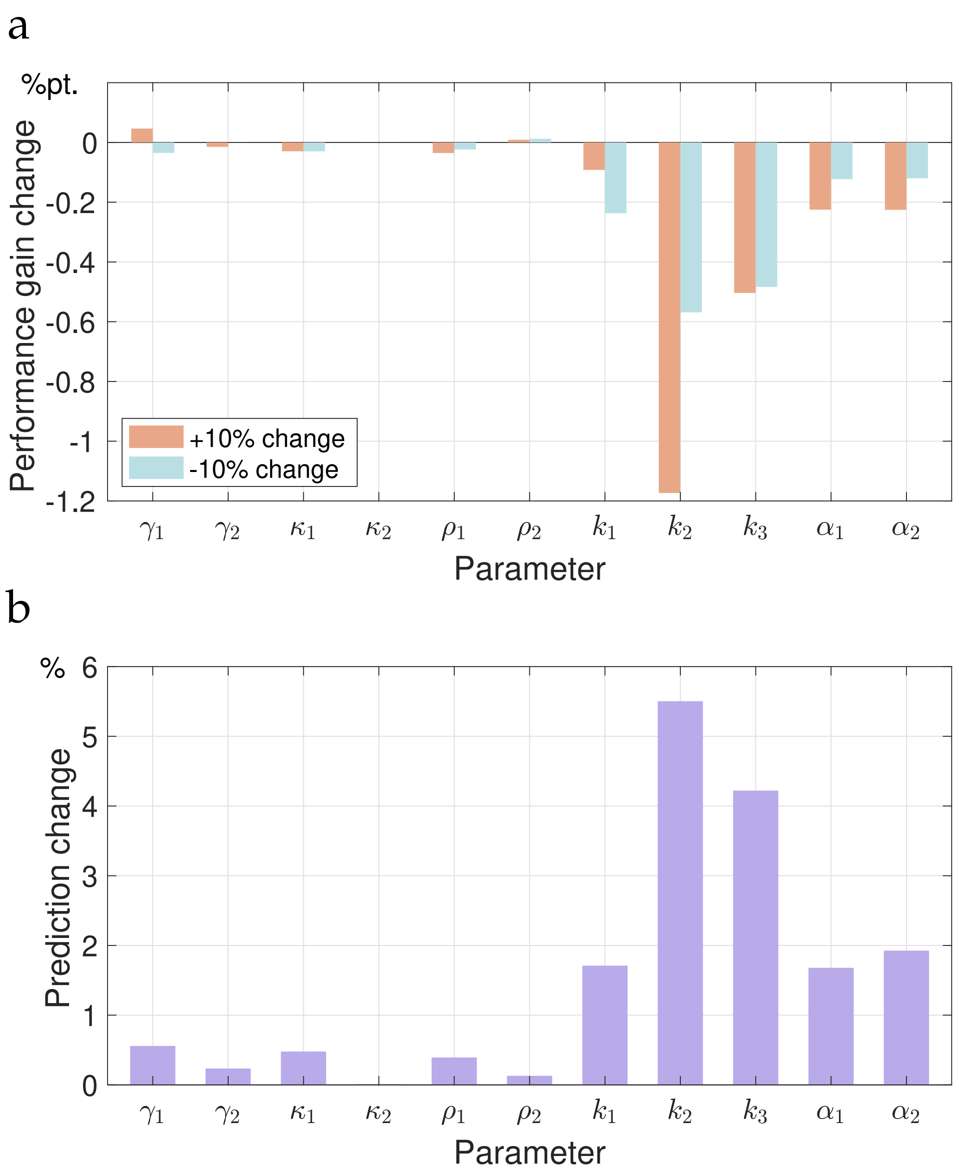

Supplementary Figure 8: Sensitivity analysis for the fitted parameters for the network model. **a:** Change in the performance gain compared to the fitted parameters, calculated over the entire data when each fitted parameter is either increased or decreased by 10% one at a time. Mostly the performance drops when parameters are varied, which is expected given that the parameters were optimized (over the training data). **b:** Average change in the 1–4-week predictions when each parameter is either increased or decreased by 10% one at a time. The relative changes are first calculated for each country, and the percentual changes are then averaged over all countries:

$$\frac{1}{2\cdot 28}\sum_{\text{pert}=1}^{2}\sum_{i=1}^{28}\frac{\sum_{t=1}^{499}\sum_{\tau=1}^{4}\left|\hat{y}_i^{(\text{pert})}(t+\tau|t)-\hat{y}_i(t+\tau|t)\right|}{\sum_{t=1}^{499}\sum_{\tau=1}^{4}\hat{y}_i(t+\tau|t)},$$

where $\hat{y}_i^{(\text{pert})}(t+\tau|t)$ denotes the τ-week ahead prediction at time t, with a perturbed parameter. The sum over pert $= 1, 2$ corresponds to either an increase or decrease in the parameter value. Note that σ_β is excluded from the sensitivity analysis, since it was chosen as the grounding variable for all the other covariance parameters.

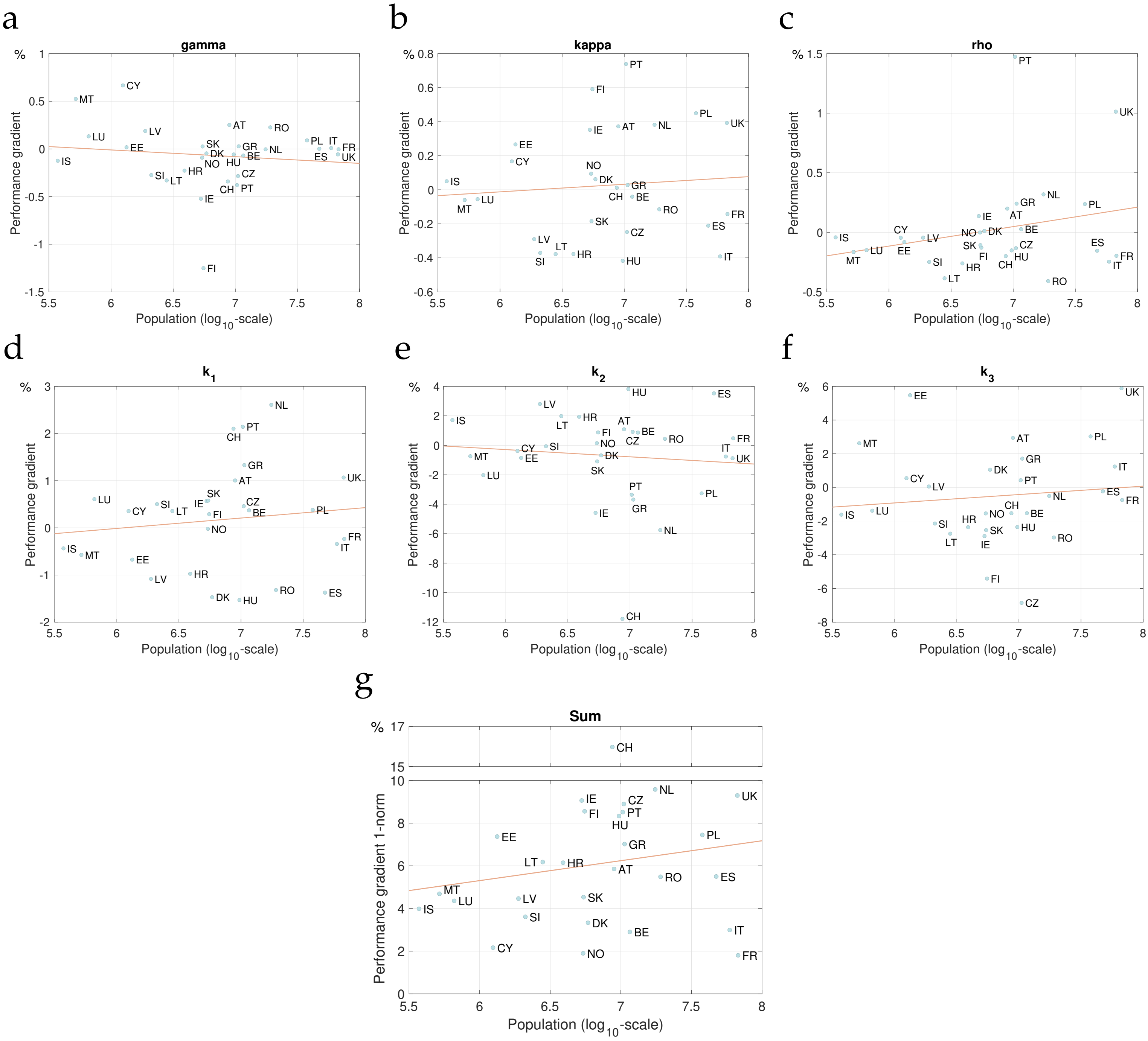

Supplementary Figure 9: Country-specific sensitivity analysis. **a–f:** Different parameters (or parameter groups) were increased or decreased by 10%, and the normalized performance difference $\left(E_i^-(\mathbf{T}) - E_i^+(\mathbf{T})\right)/E_i^{(\mathrm{ref})}(\mathbf{T})$ was calculated over the entire dataset ($\mathbf{T} = \{1, \ldots, 499\}$) for each country i; the $\pm$ superscript indicates a parameter increase or decrease. A positive value means that an increase in the corresponding parameter value would improve performance for country i. Regression lines are plotted to indicate trends depending on population size. **a:** γ_1 and γ_2 changed simultaneously ($R^2 = 0.016$ for the regression line). **b:** κ_1 and κ_2 changed simultaneously ($R^2 = 0.007$). **c:** ρ_1 and ρ_2 changed simultaneously ($R^2 = 0.066$). **d:** k_1 changed ($R^2 = 0.016$). **e:** k_2 changed ($R^2 = 0.009$). **f:** k_3 changed ($R^2 = 0.012$). **g:** Sum of the absolute values of the sensitivities with respect to all parameters. The higher the value, the larger the improvement that could be achieved by fitting country-specific parameters ($R^2 = 0.035$).

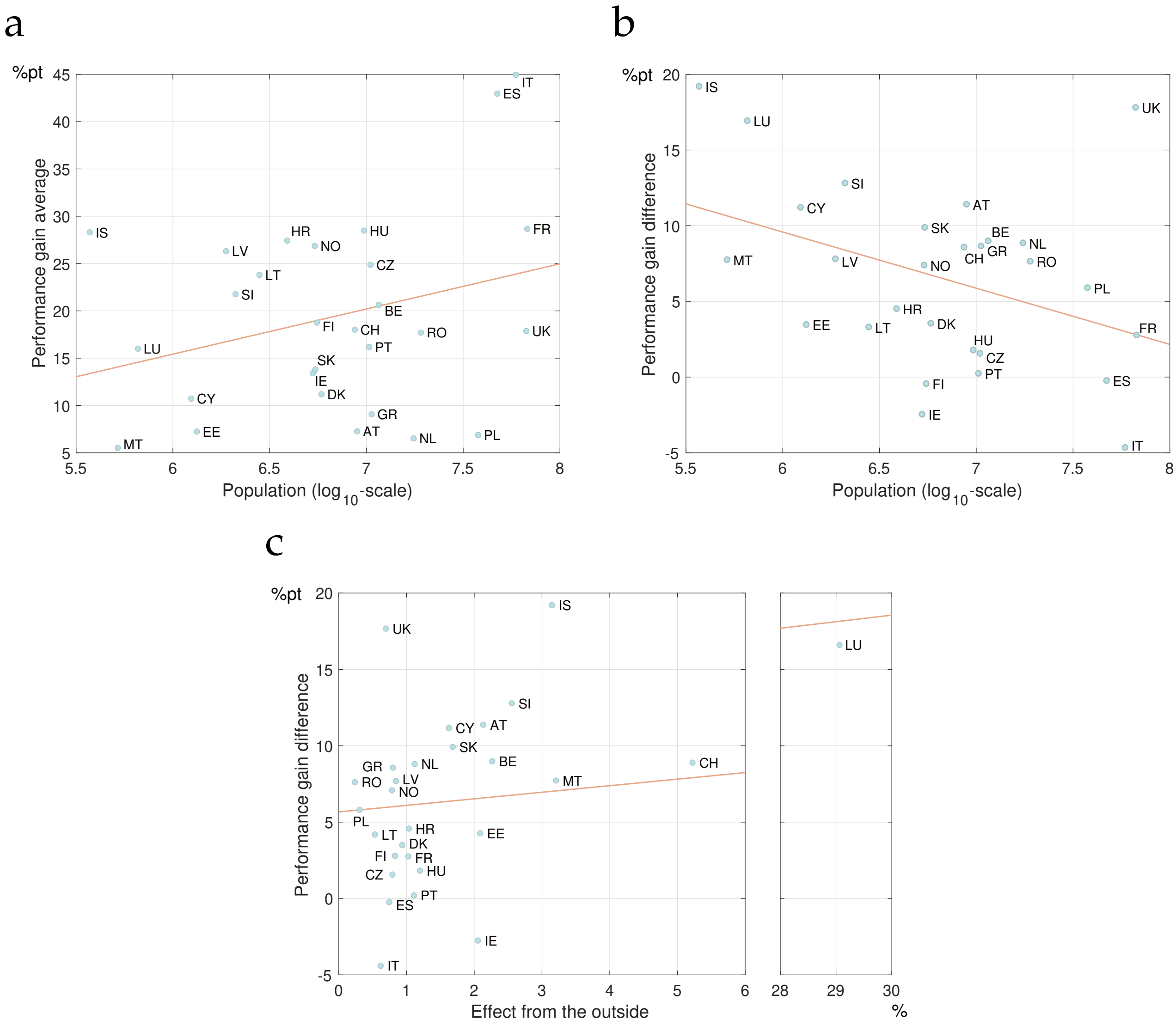

Supplementary Figure 10: **a:** Average of the performance gains of the network model and isolated models for each country, plotted against their populations (in log-scale). The linear regression line has $R^2 = 0.084$, although it is entirely driven by the good performance for Italy and Spain ($R^2 = 0.0002$ without them). **b:** Difference between the performance gains of the network model and isolated models for each country, plotted against their populations (in log-scale). The linear regression line has $R^2 = 0.151$. The R^2 increases to 0.331 if UK is excluded (in any case, the data for UK cover only one epidemic season 2023–2024 and is expected to deviate from the other results). **c:** Difference between the performance gains of the network model and isolated models for each country, plotted against the percentual effect from outside (see Fig. 2c in the main text). The linear regression line has $R^2 = 0.158$.
