# Supplementary material for: Networked SIRS model with Kalman filter state estimation for epidemic monitoring in Europe
Source: Commun Med (Lond). 2026 May 8;6:398. doi: 10.1038/s43856-026-01611-9 (PMC13376820; doi:10.1038/s43856-026-01611-9)
Supplement: Supplementary file 1 — Transparent Peer Review file [file 43856_2026_1611_MOESM1_ESM.pdf]

nature portfolio

Peer Review File

Networked SIRS model with Kalman filter state estimation for epidemic monitoring in Europe

Corresponding Author: Dr Atte Aalto

This file contains all reviewer reports in order by version, followed by all author rebuttals in order by version.

Version 0:

Reviewer comments:

Reviewer #1

(Remarks to the Author)
See attached review.

Reviewer #2

(Remarks to the Author)

The study by Aalto and collaborators evaluates the use of a network-based metapopulation model, integrated with an Extended Kalman Filter (EKF), for forecasting influenza-like illness (ILI) in Europe across multiple seasons. The model incorporates both local disease data and mobility data between countries to improve forecasting accuracy. The authors assert that the network model was found to outperform isolated and mean-field models. This suggests that the integration of mobility data significantly enhances the model's performances and provides more interpretable results.

The article is well-written and easy to read despite the large (and necessary) amount of highly technical detail, the data are impressive, and the Kalman approach is very interesting. However, I am curious whether the question the authors address is truly compelling. When analyzing the spread of a disease across a continent, the most critical question is determining the appropriate spatial granulometry to use, rather than comparing the contribution of mobility when using a very coarse granulometry, such as that of the country. This is particularly noteworthy, as the authors emphasize that the contribution of travelers to the spread of flu is less than 5% (see page 3). Therefore, a comparison of a model at country scale with mobility versus a model without mobility at the NUTS1 or NUTS2 scale would have been more suitable.

I have additional remarks and comments to share. First, when comparing results from different models, it's important to take into account the number of parameters in each model. It is essential to ensure that the optimal model does not possess a significantly higher number of parameters compared to the least effective model. An increase in the number of parameters leads to a corresponding increase in the degrees of freedom and flexibility, which facilitates more precise adjustments to the data. This approach is a common one in the field of statistics. For instance, it is employed in AICs and adjusted R-squares.

The main drawback of the approach employed in this work is, for me, the use of the homogeneous mixing for very large population. As there are many countries with populations exceeding 30-50 million, it is an unrealistic hypothesis to expect a homogeneous mixing between people.

Another significant issue is the use of multiple tuning parameters and certain “magic numbers” such as K (see page 20). Apart from the issues of non-identifiability that this entails, it suggests a lack of rigor which may mask significant calibration problems. A sensitivity analysis is necessary to assess the impact of these tuning parameters and magic numbers.

The Supplementary Table 2 includes information on the values of these tuning parameters. However, the authors do not provide values for other parameters such as \phi or \mu... These values are important for comparisons between countries and for comparison with the literature. Considering \beta_i(t), equation (4) is particularly illustrative for the important problem of non-identifiability of a model. In eq (4), we observe a succession of products between different parameters, making the model structurally non-identifiable. For instance, if we expand eq (4), we obtain the term k_1*(1-k_2)*k_3*\mu. Furthermore, we may inquire about the rationale behind the link between the transmission rate and the recovery rate. What is the

explanation for this phenomenon? Could you please provide any references for this?

Considering the Kalman filter, the authors use the EKF, which is not defined in Kalman's original paper [45]. Consequently, it is necessary to refer to an alternate reference. Furthermore, references regarding the application of EKF in epidemiology invariably pertain to papers employing the Ensemble Kalman Filter, which is a Monte Carlo version of the Kalman Filter. I strongly recommend utilizing the following references for EKF in epidemiology: Cazelles & Chau (1997), Using the Kalman filter and dynamic models to assess the changing HIV/AIDS epidemic. Mathematical biosciences, 140; Kalivianakis et al (1994). Reconstruction of the seasonally varying contact rate for measles. Mathematical biosciences, 124.

In addition, to determine the relevance of the EKF, we need to visualize specific temporal evolutions of \beta, at least for some country. It is essential to ensure that these temporal evolutions of \beta do not perfectly coincide with those of y_i(t), but rather precede them by several weeks.

In conclusion, while the authors' work is intriguing, I question the relevance of the question it addresses. When studying the spread of a pathogen on a large scale, it is essential to determine the most relevant spatial granulometry. In any case, to facilitate a more comprehensive understanding of the approach, it is essential to clarify numerous technical details (see below).

Other comments

Abstract, the first sentence: “Metapopulation models, which consider epidemic spread across interconnected regions, can provide more accurate epidemic predictions compared to country-specific models.” OK, but can you clarify whether this is due to accounting mobility or the use of a metapopulation with smallest population sizes?

Fig 1c and last paragraph page 3: This appears to be an effect of the population size. This issue must be addressed.

Page 4, Mean-field approximation: I would like to know about the differences between this model and the gravity model proposed by the Grenfell group in the 2000s.

Page 4: simplify eq (4)

Page 5 and after: It's not clear whether the observations are incidence or prevalence.
Typically, the data available is incidence, whereas the equation used for y_i(t) is prevalence.

Fig 4: The results are somewhat exaggerated. The network model performs almost as well as the mean-field model.

Fig 6: Idem

Page 11: About the adoption of a similar framework for other diseases, I believe that this would present a more substantial challenge in the case of vector-borne diseases, such as dengue.

Page 14: More explanations are needed for the \kappa parameters.

Page 15: Differences between eq (4) and eq (9)?

Page 15 and after: It is very important that you could clarify if there are any parameters that are identical between the mobility model and the isolated model? A Table would be nice.

Page 16: Why u_i(t) is defined as follows?

Page 16: I suggest using c_i(t) / \Delta T . \mu because “7” is not so easy to understand when reading the manuscript.

Page 17: “In order to produce probabilistic forecasts, a final calibration step will be carried out to find a proper scaling factor for the covariances.” I’m sure that it’s a key sentence but I don’t exactly understand what is done.

Page 17, Parameters fitting: Explanations about the use of the simulated annealing. Could you please clarify whether the EKF is run for each iteration of the simulated annealing?

Page 19: redefined “28”.

Supplementary Table 1: \gamma_1 and \gamma_2 is defined but not \gamma for eq (3).

Supplementary Table 2: There are values for the tuning parameters but not for the parameter of the epidemiological model. These values are important for comparisons between countries and for comparison with the literature.

Supplementary Fig 1: We need population density and not surface area!

Version 1:

Reviewer comments:

Reviewer #1

(Remarks to the Author)
I am very satisfied with the authors responses and recommend publication.

Reviewer #2

(Remarks to the Author)

The authors have clearly invested a considerable amount of time and effort into refining their manuscript. The revisions have significantly enhanced its clarity, making it far more accessible and easier to follow. Additionally, the technical details have been carefully revised. This thorough attention to detail has greatly improved the overall quality of the manuscript.

However, I remain somewhat unconvinced of the importance of mobility between states with vastly different population sizes in the context of flu epidemics. This skepticism is reinforced by the authors' statement on page 5, where they note that people who travel account for less than 5% of influenza infections. Additionally, the parameters modeling mobility between states (the \gamma_i) appear to be relatively insensitive, which further weakens the argument for their critical role. While I believe this work is certainly worth publishing, I would suggest a slightly less emphasized focus on the mobility results, especially considering that the 2- and 4-week prediction capabilities are not particularly striking. I also suspect there may be practical identifiability issues arising from the limited information available in the data. For instance, it is difficult to determine the susceptibility of populations to the seasonal flu at the start of each season, given the variations in their immunological status following prior epidemics.

In addition, I believe that the EKF could benefit from enhancements:
- It would be useful to discuss whether the complexity of U is truly necessary for the filter's performance and for improving the results.
- \sigma_{\beta} is fixed. Its chosen value is not explained, and we do not know its sensitivity. This parameter directly affects the process noise covariance matrix Q (as shown in Eq. 8). From my experience with Kalman filters, adjusting Q can have a significant impact on the filter's gain and, consequently, on the correction of state estimates. A larger value of Q leads to more large corrections of predicted state values, while a smaller value results in more conservative corrections. In my view, it would be beneficial to allow \sigma_{\beta} be adjusted alongside other parameters during the simulated annealing.

Other- Details:

Page 6: the N_I are never defined.

Page 7: References are needed for the choice of \mu=0.06 and \psi=0.0116.

Page 10: T_{max} not defined

Page 13: Sorry I don’t understand the role of K.

Page 18: “The Kalman updates are therefore mainly focused on S and I, while the updates of β are fairly small.”: OK but it’s also depend on the values in Q, including \sigma_{\beta}.

Page 18: Nice paragraph on identifiability, congratulations.

Page 20: “It can be observed (Fig. 5a and Supplementary Fig. 9) that the performance is on average slightly better for countries with larger population than with smaller population, but the performance improvement of the network model compared to isolated models is generally smaller for larger populations. …”: another possibility to consider is that random components (Q and U) may not always be a suitable fit for different demographics.

Version 2:

Reviewer comments:

Reviewer #2

(Remarks to the Author)
I have carefully reviewed the revised version of Alto et al’s manuscript. While I remain somewhat unconvinced by the authors’ interpretation of the role of mobility, I find that arguments not directly supported by the content of the manuscript,

such as the statement that “our network model has been included in the RespiCast platform”, are of limited relevance to the scientific contribution of the study.
That said, the authors have made substantial efforts to address the comments and improve the manuscript. In my view, the paper is now suitable for publication in Communications Medicine.

Open Access This Peer Review File is licensed under a Creative Commons Attribution 4.0 International License, which permits use, sharing, adaptation, distribution and reproduction in any medium or format, as long as you give appropriate credit to the original author(s) and the source, provide a link to the Creative Commons license, and indicate if changes were made.
In cases where reviewers are anonymous, credit should be given to 'Anonymous Referee' and the source.
The images or other third party material in this Peer Review File are included in the article’s Creative Commons license, unless indicated otherwise in a credit line to the material. If material is not included in the article’s Creative Commons license and your intended use is not permitted by statutory regulation or exceeds the permitted use, you will need to obtain permission directly from the copyright holder.
To view a copy of this license, visit https://creativecommons.org/licenses/by/4.0/

Response to reviewers

We thank the editor and the reviewers for their useful feedback, which we have duly answered below and incorporated in the text, when enhancing the clarity and correctness of the manuscript. Our responses and edits are in blue.

Please also note that we have rearranged the structure of the manuscript. Our original submission was transferred from another journal of the Nature family, which required a different organization of the manuscript where the Methods section appeared at the end of the manuscript. We have now revised the structure to follow the format of *Communications Medicine*, where the Methods section appears more prominently between the Introduction and the Results sections. In the original submission, the first two subsections in Results gave a brief overview of the network generation and the overall methodology; instead, the new organization has allowed us to integrate the former sections 2.1 and 2.2 into the Methods section and thereby streamline the presentation. Some changes done due to this integration are not marked with blue font.

We have also changed the order of the Methods subsections, so as to improve the flow of the text. For example, the ”Performance evaluation” subsection was moved before the “Parameter fitting” subsection, for consistency with the order in which the cost function used for parameter fitting is defined. Similarly, the “Calibration” subsection now appears immediately after the “Parameter fitting” subsection, to discuss the necessity of calibration early on and subsequently describe it. The text is thus more fluent - which addresses one of the reviewers’ suggestions.

We have also added the Supplementary Figure 3 that extends the explanation of the adaptive estimation of the c(t) parameters, while allowing to shorten slightly the corresponding part in the Methods section.

Reviewer 1

1 Paper Summary

The authors consider a meta-population that incorporates mobility data on land-based movement, cross-border commuting, and air and ferry travel. This model is a discrete-time SIRS model with innovations: an Extended Kalman Filter for updating parameters over time and determining uncertainties, and a network that summarizes the mobility data. The authors show percent gain improvement over a more naive model that considers cross-border transmission (but not explicit mobility data) and a model that ignores disease transmission between countries.

2 Strengths

This paper is enjoyable to read and makes a compelling case for incorporating the mobility data. I found the way that the authors incorporated the network into the Kalman filter and into the compartmental model to be interesting to think about. Especially as more data become

available on the post-pandemic landscape, I am sure that this model will be practically useful for future applications.

Response: We thank the Reviewer for the appreciation of our work.

3 Points of criticism

1. Overall, I felt that this paper could use more cross-referencing. Things like specifics on parameter selection and specifics on error calculations are perhaps appropriately kept in the Methods section and in the supplement, but a sentence stating early on that this is the case may help readers like myself.

   Response: The new organization of the manuscript (Introduction-Methods-Results), on top of being compliant with the format of *Communications Medicine*, addresses this comment by better streamlining the text. We have also labeled equations defining performance metrics and we refer to these equations in the Results section.

2. For the regional analysis, there are instances where the isolated models out-performed both the mean-field and network models (ES and IT). It would be helpful to know what happened in these instances; this may improve future iterations of the model.

   Response: We have added Supplementary Fig. 9, where we plot the performance difference of the network and isolated models as a function of the countries' populations. We can observe that in general, for larger countries the performance difference is smaller. In larger countries, data are averaged over large populations, which improves data regularity. Indeed, data for Spain and Italy are quite regular, and so these countries have the highest performance gains over the reference forecast. The inclusion of mobility effects by the network model does not improve the already very good forecasts. However, large countries may also have large regional differences, and more targeted forecasts could be more useful, if regional data were available. We refer to Supplementary Fig. 9 in Section 3.1, and we have also extended the Discussion section by a discussion on the model granularity.

3. The missing at random assumption should be justified. How typical is it for there to be random days for which all data are missing? I'd think it be more likely to have day-varying amounts of under-reporting, but to still have some amount of reporting for all days (or weeks). The imputation application of the network is interesting, but it is insufficiently justified for this application.

   Response: Indeed, instances with completely missing data are rather rare. However, when new weekly data are released, data for some countries are typically missing from the ECDC database, due to late reporting from local authorities or practical issues. In most cases, these data are then reported retrospectively within the next few weeks. However, quantifying performance for such data delays is rather complicated, which is why we used the simple "missing at random" scenario for performance comparison. We have extended the text in the beginning of Section 3.2 to explain this.

4. The section on probabilistic forecasts would benefit from a more robust analysis. Instead of looking at the long-term forecasts, I would be more interested in something like a Weighted Interval Score or an analysis of statistical coverage over the course of several epidemic seasons.

   Response: We used statistical coverage plots to tune the noise factor K as described in Section “Calibration for probabilistic forecasts”. The coverage plots are in Supplementary Fig. 5. We have added the term “coverage plots” to the caption of Supplementary Fig. 5, and we also refer to this figure in Section 2.10. In addition, we have replaced the shown long-term forecast for Switzerland by a box plot showing the averaged weighted interval scores for the 1-4-week ahead forecasts. This is broken down to individual box plots for each epidemic season shown in Supplementary Fig. 6.

4 Smaller points, and line-by-line comments

1. (Figure 1a) The map of Europe is way too small for a reader to see all the blue and red arrows.

   Response: We have increased the size of the map and the text font sizes in the map.

2. (Figure 1b) It might be interesting to visualize the matrix G for the mean-field approximation as well. There are several cases later where these models perform similarly; I wonder how alike these transition matrices are. One could also do a distance matrix on the physical distance between the countries as reference (and to show that this does not provide the same information as the mobility data).

   Response: We have included the heatmap for the matrix G of the mean-field network in Supplementary Figure 2, together with the heatmap of the adjacency matrix for the so-called gravity model network, where the network link strengths depend on the populations of the respective countries and on the inverse of the distance between the countries. We also included a heatmap showing the differences between our travel-based network and the gravity network matrices. The gravity network partially coincides with our travel-based network, but it under-estimates connections with popular holiday destinations, for example, that are captured by the flight data. This observation, which is useful to decide on the usage of gravity models, is also discussed in Sec. 2.1

3. (p.3 eq. (1)) According to the supplement and the Modeling section, you fit these γ parameters (and several other tuning parameters throughout the paper) with simulated annealing. Would you (perhaps in the supplement) give an overview of what that is?

   Response: We have added Supplementary Note 3, where the parameter fitting procedure is explained in more detail including a pseudocode for the used simulated

annealing procedure. We refer to this note in the “Parameter fitting” subsection, from where some details were moved to the new Supplementary Note.

4. (p.3) Is the normalization by the maximum of each row done just for the visualization, or for the actual matrix G used for the model? Please make this clear.

   Response: Normalisation is done for visualisation purposes only, since otherwise rows corresponding to smaller countries would be completely white. This is now clarified in the text and the caption of Figure 2.

5. (p.4 eq. (4)) Is the Gaussian noise dependent on the current value of β? Does it change over time?

   Response: The variance of the noise is constant over time. This is now mentioned after Eq. (7) (formerly Eq. (4)).

6. (Figure 2) The SIRS model is labeled as “Stochastic,” but the ODEs that are written are not the stochastic forms.

   Response: Since the Kalman filter prediction step in fact uses the deterministic model, we have removed the word “Stochastic” from Fig. 1 (former Fig. 2). The stochastic model is used for the calculation of the Kalman gain and for the probabilistic forecasts.

7. (p.5) “−parameter” I assume is a typo.

   Response: Yes, it should be \beta-parameter, and it is now fixed.

8. (Figure 6a) I had difficulties understanding how the error was calculated for this plot. More details around where it is mentioned in the text (p.8) would be helpful.

   Response: We have included the definition of the reconstruction error into the text. We have also labeled the equation where the forecast error is defined and we refer to this equation in the Results-section.

9. (p.10) κ1 is used at the beginning of Section 2.6, but it was not introduced before this.

   Response: With the new manuscript structure, κ1 is introduced in the Methods section before Section 3.4 (formerly Section 2.6). To improve readability, we still describe κ1 as “noise scaling factor” in Section 3.4 and refer to Eq. (6), where the parameter κ1 appears.

5 Recommendation

After that my above comments are addressed fully, I would recommend publication. The statistical analysis is robust (although the analysis on the probabilistic forecasts is wanting).

This paper is already interesting and I believe after some modifications it would make an appropriate article for Communications Medicine.

Response: We thank the Reviewer for all the constructive suggestions, which we have carefully and thoroughly addressed, and for the appreciation for our work.

Reviewer 2

The study by Aalto and collaborators evaluates the use of a network-based metapopulation model, integrated with an Extended Kalman Filter (EKF), for forecasting influenza-like illness (ILI) in Europe across multiple seasons. The model incorporates both local disease data and mobility data between countries to improve forecasting accuracy. The authors assert that the network model was found to outperform isolated and mean-field models. This suggests that the integration of mobility data significantly enhances the model's performances and provides more interpretable results.

The article is well-written and easy to read despite the large (and necessary) amount of highly technical detail, the data are impressive, and the Kalman approach is very interesting.

Response: We thank the Reviewer for the appreciation of our work.

However, I am curious whether the question the authors address is truly compelling. When analyzing the spread of a disease across a continent, the most critical question is determining the appropriate spatial granulometry to use, rather than comparing the contribution of mobility when using a very coarse granulometry, such as that of the country. This is particularly noteworthy, as the authors emphasize that the contribution of travelers to the spread of flu is less than 5% (see page 3). Therefore, a comparison of a model at country scale with mobility versus a model without mobility at the NUTS1 or NUTS2 scale would have been more suitable.

Response: We agree with the reviewer that another interesting question, which is complementary to our own, is the assessment of the spatial granularity that improves model predictions. However, we focus on the question of how to best account for mobility information when performing epidemic predictions based on available data with given granularity. In particular, our forecasting task is based on ECDC's data, available and curated on the Respicast platform, where country-specific forecasts are requested, based on country-specific aggregated data. The chosen granularity is dictated by the availability of data (both travel data for network construction and the ILI incidence data) that are collected at EU level. This collection methodology is also representative of efforts conducted in other countries, where data pooling is performed at a spatial scale that is decided by health authorities and by the availability of sampling stations (see e.g. refs [26,27,28]).

While some regional data could be available in country-specific sources, it is not the case for all included countries. Moreover, there is no such a curated and vast database including data at various granularities for the same geographical area, which would allow a cross-scale systematic comparison of forecasting performance. A related question, when the goal is to

forecast epidemic development in one country, would be if it is better to pool regional data together before integrating it to a forecasting method, or to make first regional forecasts, and then pool the forecasts. This would be a first step in this direction. Accounting for the various angles of the problem, we now discuss the topic of optimal granularity in the Discussion, leaving it to future studies which may address it using our approach and other datasets.

I have additional remarks and comments to share. First, when comparing results from different models, it's important to take into account the number of parameters in each model. It is essential to ensure that the optimal model does not possess a significantly higher number of parameters compared to the least effective model. An increase in the number of parameters leads to a corresponding increase in the degrees of freedom and flexibility, which facilitates more precise adjustments to the data. This approach is a common one in the field of statistics. For instance, it is employed in AICs and adjusted R-squares.

Response: It is true that the different models have different numbers of fitted parameters. The network model has 11, the mean-field model has 10, and the isolated models have 8 fitted parameters. To foster a fair comparison despite this difference, we have followed a computer science approach by splitting the data into training data that is used for parameter fitting and test data that is used to confirm that the performance improvement is not just due to a more flexible model. Indeed, we observe the same performance difference both on the training data and the test data. We now explicitly state the numbers of fitted parameters for each model in the beginning of the "Parameter fitting" section. Moreover, we state this as the reason for the training/test data split both in the "Parameter fitting" subsection and Section 3.1 in the results.

The main drawback of the approach employed in this work is, for me, the use of the homogeneous mixing for very large population. As there are many countries with populations exceeding 30-50 million, it is an unrealistic hypothesis to expect a homogeneous mixing between people.

Response: Indeed, the assumption of homogeneous mixing (and homogeneous population) is a big simplification of the SIR model. This assumption is in fact never true in any population size. However, the simplicity of the model makes it well-suited for short-term projections, and for data integration schemes such as the Kalman filter. Despite its shortcomings, the SIR model (or its variants) is still the principal workhorse of epidemic modelling and is abundantly employed for forecasting, in particular when more granular data are not readily available (see e.g. the models employed in the Respicast or Influcast initiatives).

When simply looking at the incidence data of a particular country, the data tend to be at least locally reproducible by a SIR model trajectory, even if the underlying assumptions of the model development are violated. For short-term forecasting purposes, this data reproducibility is really all that matters. For the stochastic model, the assumption of perfect mixing leads to a very low level of stochasticity in the system. This is the reason why we introduce the parameter kappa1 that scales the stochastic noise. In the revision, we have extended the reasoning for the introduction of this parameter.

Another significant issue is the use of multiple tuning parameters and certain "magic

numbers” such as K (see page 20). Apart from the issues of non-identifiability that this entails, it suggests a lack of rigor which may mask significant calibration problems. A sensitivity analysis is necessary to assess the impact of these tuning parameters and magic numbers.

Response: Each tuning parameter was included in our pipeline for a precise scope - either mathematical or to improve the interpretation of the results. Given that the reasoning for the addition of each tuning parameter was not entirely clear, we have now clarified it in the text. The networks G1 and G2 are in principle given as actual travel fluxes, but it is uncertain how much each cross-border trip influences the epidemic state of the respective countries, since durations of trips, risk factors associated with traveling etc. are not known. Due to these unknown factors, gamma_1 is regarded as a tuning parameter. The third network component G3 is approximated by population densities, and it therefore must be scaled (by an unknown parameter) in order to be included. As we explained, the uncertainties arising from the Langevin equation derivation as well as the binomial distribution for observations are seriously under-estimating the variances of the noise processes, warranting their scaling by tuning parameters. In addition, keeping the variances as tuning parameters allows the parameter fitting procedure to find the balance in the Kalman filter updates done to the epidemic state (S,I) and the transmission parameter beta. The state-independent noise terms (parameters kappa2 and rho2) are the only terms that are included for more technical reasons to prevent degenerate noise terms during times of low incidence, improving responsiveness at the onset of a new wave.

Sensitivity analysis has been carried out for all fitted parameters. Results are shown in Supplementary Figure 8 using the parameters fitted for the network model. The sensitivity with respect to the parameters kappa2 and rho2 is very small, but the values of these parameters are very small in the network model.

As we already discussed in Section 3.4 (formerly 2.6), the gamma_2 parameter does not seem to be very well identifiable. This is observed both by the sensitivity analysis, as well as the fact that its value changes considerably when the amount of data used for fitting is varied. However, lack of sensitivity/identifiability is only a problem if one wishes to make conclusions based on the parameter values, while our focus is on forecasting performance.

The Supplementary Table 2 includes information on the values of these tuning parameters. However, the authors do not provide values for other parameters such as \phi or \mu... These values are important for comparisons between countries and for comparison with the literature.

Response: All parameters are now included in Supplementary Table 2.

Considering \beta_i(t), equation (4) is particularly illustrative for the important problem of non-identifiability of a model. In eq (4), we observe a succession of products between different parameters, making the model structurally non-identifiable. For instance, if we expand eq (4), we obtain the term k_1*(1-k_2)*k_3*\mu.

Response: There is no structural non-identifiability in Eq. (7) (formerly Eq. (4)). There are three parameters k_1, k_2, and k_3, that are multiplying three different components inside the square brackets (in different combinations). The terms inside the square brackets form

the difference between a reference value for beta (the first two terms) and the current value (the third term). Such a term is pushing beta_i towards the reference value and k_1 is the strength of this push. The reference value is a linear combination of two values, the weighted average of beta_j's of the network neighbors of the country i, and a constant value k_3*\mu that acts as a “grounding value”, that is, a value towards which beta_i's would converge in the absence of data for an extended period of time. The parameter k_2 weighs the contribution of these two terms in the reference value, since one of them is multiplied with k_2 and the other with 1-k_2. Each of these three parameters has got an individual interpretation in the model and they never appear in an unidentifiable way. In fact, based on the sensitivity analysis, these three are among the best identifiable parameters. We have extended the explanation of these parameters when they are first introduced in Eq. (7).

Furthermore, we may inquire about the rationale behind the link between the transmission rate and the recovery rate. What is the explanation for this phenomenon? Could you please provide any references for this?

Response: There is no link between the recovery rate and transmission rate per se, and there would be no difference if \mu was left out from Eq. (7) (formerly Eq. (4)), since it only acts as a scaling for the fitted parameter k_3. The reason for this scaling is to give a clear interpretation to k_3. The reproduction number R0 in a SIR model is the ratio beta/mu, and so the R0 corresponding to the baseline value beta=k_3*mu is k_3. We now mention this connection when introducing the beta-parameter dynamics.

Considering the Kalman filter, the authors use the EKF, which is not defined in Kalman's original paper [45]. Consequently, it is necessary to refer to an alternate reference. Furthermore, references regarding the application of EKF in epidemiology invariably pertain to papers employing the Ensemble Kalman Filter, which is a Monte Carlo version of the Kalman Filter. I strongly recommend utilizing the following references for EKF in epidemiology: Cazelles & Chau (1997), Using the Kalman filter and dynamic models to assess the changing HIV/AIDS epidemic. Mathematical biosciences, 140; Kalivianakis et al (1994). Reconstruction of the seasonally varying contact rate for measles. Mathematical biosciences, 124.

Response: It is true that the EKF is not introduced in Kalman's original work, but the generalisation is rather straightforward, and there is no “canonical” reference for the EKF. We have included a reference to the textbook “Optimal filtering” by Anderson and Moore. We have also included the two suggested references to EKF in epidemiology. We have slightly reworded the text in the introduction to include different Kalman filter versions.

In addition, to determine the relevance of the EKF, we need to visualize specific temporal evolutions of \beta, at least for some country. It is essential to ensure that these temporal evolutions of \beta do not perfectly coincide with those of y_i(t), but rather precede them by several weeks.

Response: We have added Supplementary Fig. 4 visualizing the beta(t) estimate for Latvia for three epidemic seasons 2016-2019, including the season for which forecasts are shown in Fig. 4. There we can clearly see how the network model and mean-field models are able to anticipate the upcoming wave of two epidemic seasons where Latvia is somewhat behind

the European average. However, since the predictions are completely based on data, beta-estimate cannot increase before anything is visible in the incidence data.

In conclusion, while the authors' work is intriguing, I question the relevance of the question it addresses. When studying the spread of a pathogen on a large scale, it is essential to determine the most relevant spatial granulometry.

Response: As discussed above, our focus is on the effect of including mobility into a model with given spatial granularity. We believe that our observations are of interest regardless of the chosen spatial granularity, as they address the improvement of predictions with available data. We do agree with the reviewer that the question on the effect of granularity is interesting but somewhat orthogonal to our question, and perhaps will be addressed in future works; given the interest for the community, we nonetheless discuss it in the Discussion.

In any case, to facilitate a more comprehensive understanding of the approach, it is essential to clarify numerous technical details (see below).

Other comments

Abstract, the first sentence: “Metapopulation models, which consider epidemic spread across interconnected regions, can provide more accurate epidemic predictions compared to country-specific models.” OK, but can you clarify whether this is due to accounting mobility or the use of a metapopulation with smallest population sizes?

Response: The network model introduced in the manuscript has the same granularity as the country-specific models, and since evaluations are made at the country-level, the difference in forecasting performance stems completely from connecting the regions by the mobility network. We have slightly reworded this sentence in the abstract, since it was somewhat mixing the words “region” and “country”. Indeed, in our model, the interconnected regions are the countries included in ECDC’s Respicast initiative.

Fig 1c and last paragraph page 3: This appears to be an effect of the population size. This issue must be addressed.
Response: We interpret this comment to be about Luxembourg appearing as an outlier. Small population combined with a high number of cross-border workers makes Luxembourg an exception in this comparison, but it is not an error to be fixed, nor an indication of the fact that population size alone makes a difference: the relative proportion of internal vs. external contribution to contagion is country-specific and depends on the amount of daily travellers across the borders, including cross-border workers. In this regard, Luxembourg enjoys a special standing, with which many of the authors, being based in Luxembourg, are well familiar.

Page 4, Mean-field approximation: I would like to know about the differences between this model and the gravity model proposed by the Grenfell group in the 2000s.

Response: The gravity model assumes that the influx from region *j* to region *k* is proportional to $N_k^{\tau_1} I_j^{\tau_2} / d_{jk}^{\rho}$ where d_{jk} is the physical distance between the communities *j* and *k* and τ_1, τ_2, and ρ are tuning parameters. The parameters τ_1, τ_2 account for travel dispersion between communities of different sizes. This "gravity network" would be an alternative way to construct the network between regions. In terms of complexity, it is somewhere in between our travel-based network and the mean-field network that simply incorporates the average incidence across all included regions into the dynamics of each individual region. In Grenfell's work, the gravity network is used to couple smaller communities in England and Wales. We have included Grenfell's article introducing the gravity model as a reference on metapopulation models in the Introduction and there we point out that the network is based on distance. In addition, by way of comparison, we have added a heatmap of the gravity model in Supplementary Figure 2. While there is some overlap, the networks are still quite different: for instance, the gravity model underestimates long-range connections due to popular flight trips, while overestimating connections for countries such as Italy or Poland.

Page 4: simplify eq (4)

Response: It is unclear what the reviewer means by "simplify". As explained above, the term inside the square brackets has a clear meaning, that is, it represents a difference between a reference value and the current value of beta_i. Opening the parentheses would therefore hardly clarify Eq. (7) (formerly Eq. (4)).

Page 5 and after: It's not clear whether the observations are incidence or prevalence. Typically, the data available is incidence, whereas the equation used for y_i(t) is prevalence.

Response: It is true that I(t) is prevalence and the data are incidence. The output is obtained as y = c*I, where c modulates the share of observed cases out of all infections. As we remarked in the manuscript, in our earlier implementations of the Kalman filter on epidemic models (the CoWWAn pipeline and the previous Respicast season), we used a more complex observation model containing an additional state variable that was keeping track of daily/weekly new cases. This output corresponded precisely to disease incidence, but with a fairly strict implicit assumption that all observations were infections that had occurred on the same day/week. In contrast, our current, simpler observation model assumes that an infection can be detected and counted at any time when the infected individual is in the I-compartment. We noted that performance was just as good with the more simple observation model where y is obtained directly from the I-compartment size, and we therefore opted for the simpler model. We have extended this remark (at the end of the "Extended Kalman filter implementation" section) to specifically point out this discrepancy between incidence and prevalence. We anyway have the scaling coefficient y(t) = c(t)*I(t) which tunes the magnitudes of the different entities.

Fig 4: The results are somewhat exaggerated. The network model performs almost as well as the mean-field model.
Fig 6: Idem

Response: In the captions, we mainly compared the network model to the isolated models. We have revised the captions to include also the mean-field model and we have clarified the significance of the results associated with each figure.

Page 11: About the adoption of a similar framework for other diseases, I believe that this would present a more substantial challenge in the case of vector-borne diseases, such as dengue.

Response: We agree that vector-borne diseases may induce additional challenges in predictions, as well as in model and data handling. Nonetheless, previous literature results (Zeng et al, 2023) support the possibility to use metapopulation models and Kalman filters also for such diseases - although currently for smaller areas. While the underlying equations should be adjusted accordingly, our results encourage the further investigation and adoption of such frameworks. The Discussion now embeds such remarks.

Page 14: More explanations are needed for the \kappa parameters.

Response: The derivation of the stochastic Langevin equation implicitly assumes a well mixed homogeneous population. This leads to severely underestimated noise levels. We therefore introduce scaling of the noise by kappa_1, while the noise modulation by the square root of the transition rate is kept. We have extended and clarified the introduction of the kappa parameters.

Page 15: Differences between eq (4) and eq (9)?

Response: Following the new organization of the manuscript, the equation for the beta-parameter dynamics now appears only once to avoid confusion.

Page 15 and after: It is very important that you could clarify if there are any parameters that are identical between the mobility model and the isolated model? A Table would be nice.

Response: We have now also included the fixed parameters to Supplementary Table 2. They include the transition rates I -> R and R -> S. They are the same for all models.

Page 16: Why u_i(t) is defined as follows?

Response: It is unclear whether the reviewer means u_i (without time-dependency) or U_i(t). We have extended the explanations of both terms. The measurement variance U_i(t) consists of two terms, a state dependent term arising from the binomial distribution, and a second, state-independent term whose inclusion is more technical. It prevents the variance from approaching zero during times of low incidence. These two terms are scaled by global tuning parameters rho_1 and rho_2, as well as by country-specific factors u_i. These factors are not fitted based on performance, but they are calculated as the average ratio of the signal variance around a moving average, and the variance predicted by the binomial distribution assumption.

Page 16: I suggest using c_i(t) / \Delta T . \mu because “7” is not so easy to understand when reading the manuscript.

Response: Changed to c_i(t) * \Delta t / \mu, where \Delta t = 1/7.

Page 17: “In order to produce probabilistic forecasts, a final calibration step will be carried out to find a proper scaling factor for the covariances.” I’m sure that it’s a key sentence but I don’t exactly understand what is done.

Response: The paragraph in question has been moved to the end of the “Parameter fitting” section where it is perhaps easier to understand, as it directly precedes the section on noise calibration. In this paragraph, it is explained that the state estimate does not change if all covariances in the Kalman filter are scaled by a constant. Since the parameter fitting is done using the state estimate only, it is impossible to capture the true noise level by this fitting. However, for probabilistic forecasts, it is imperative to have a correct noise level and, therefore, an additional calibration step is required.

Page 17, Parameters fitting: Explanations about the use of the simulated annealing. Could you please clarify whether the EKF is run for each iteration of the simulated annealing?

Response: Indeed, we run the EKF for each iteration and use the 1-4-week forecasting performance as the cost function. This is now mentioned in the “Parameter fitting” section. We have also added a new Supplementary Note 2, where we explain the parameter fitting procedure in more detail.

Page 19: redefined “28”.

Response: We now mention here that the sum with i = 1,…,28 is a sum over all 28 countries.

Supplementary Table 1: \gamma_1 and \gamma_2 is defined but not \gamma for eq (3).

Response: \gamma has been added to Supplementary Table 1.

Supplementary Table 2: There are values for the tuning parameters but not for the parameter of the epidemiological model. These values are important for comparisons between countries and for comparison with the literature.

Response: We apologise for the omission. Parameters of the epidemiological model are now mentioned in the main text as well as in Supplementary Table 2. These parameters are the same for all countries and for all models.

Supplementary Fig 1: We need population density and not surface area!

Response: The numbers indicated by *D* are indeed population densities (with unit km^{-2}). This is mentioned in the caption.

Response to reviewers

We wish to thank the Editor and the Reviewers for their feedback on the first revision of our manuscript.

We are grateful to Reviewer 1 for carefully reviewing and appreciating our revised manuscript, and recommending it for publication.

Below, we give point-by-point responses to the comments provided by Reviewer 2. Our responses are highlighted in blue font. We have also provided a pdf-file of the revised manuscript where changes are highlighted. In addition to the changes suggested by the Reviewer, we have updated the structure and the formatting of the manuscript, as requested by the Editorial office, so as to comply with the journal's style requirements; to maintain readability, these formatting changes are not always highlighted. In particular, the abstract has been structured following the journal style. In the introduction, the article's results are only discussed in the last paragraph and, to streamline this last paragraph, some changes were done also elsewhere in the introduction.

Reviewer #1 (Remarks to the Author):

I am very satisfied with the authors responses and recommend publication.

Response: We thank the Reviewer for the feedback and for the positive assessment.

Reviewer #2 (Remarks to the Author):

The authors have clearly invested a considerable amount of time and effort into refining their manuscript. The revisions have significantly enhanced its clarity, making it far more accessible and easier to follow. Additionally, the technical details have been carefully revised. This thorough attention to detail has greatly improved the overall quality of the manuscript.

Response: We thank the Reviewer for the feedback and for appreciating our revision.

However, I remain somewhat unconvinced of the importance of mobility between states with vastly different population sizes in the context of flu epidemics. This skepticism is reinforced by the authors' statement on page 5, where they note that people who travel account for less than 5% of influenza infections. Additionally, the parameters modeling mobility between states (the γ_i) appear to be relatively insensitive, which further weakens the argument for their critical role. While I believe this work is certainly worth publishing, I would suggest a slightly less emphasized focus on the mobility results, especially considering that the 2- and 4-week prediction capabilities are not particularly striking. I also suspect there may be practical identifiability issues arising from the limited information available in the data. For instance, it is difficult to determine the susceptibility of populations to the seasonal flu at the start of each season, given the variations in their immunological status following prior epidemics.

Response: We agree that the mean-field model's performance is nearly as good as the mobility-network model's for mid- and long-term forecasts. Nonetheless, for surveillance initiatives aimed at informing practitioners and decision makers, even such a small improvement can be valuable; for instance, our network model has been included in the RespiCast platform[1],

[1] See the recent medRxiv preprint about RespiCast: `https://doi.org/10.1101/2025.10.30.25339155`

where it is consistently a top performer. Moreover, for the epidemic season 2024–2025, its forecast clearly outperforms those obtained through isolated models, which provides an additional external validation on completely new data. Our network model also retains significant advantages in coping with missing data, common in routine surveillance, and in identifying critical events, such as epidemic peak and wave onset. In fact, although it is true that the mobility effect would likely be stronger on a finer granularity, where the share of people traveling between regions is higher, the external contribution may still be significant for the onset of a new wave in a region during times of low incidence (such as, indeed, at the beginning of a new epidemic wave). When the regional incidence is higher, then certainly the external contribution becomes less significant. Indeed, during wave onsets the detailed mobility-network model performs better than the alternatives.

Following the Reviewer's suggestion, we have edited the Introduction and Discussion to clarify that our network model provides a relatively mild improvement of mid- and long-term forecasts (which however, as mentioned above, is still valuable for surveillance and scenario building, as testified by the Respicast initiative), but can significantly improve estimates in case of missing or incomplete data, and allow us to estimate epidemic peak and wave onsets with smaller uncertainties – all of which is important for practical epidemic management.

Parameters γ_i, accounting for the contributions of different forms of travel, are not representative of the entire role of network interconnections in our model, which also includes parameters β_i and their time evolution. In the dynamics of parameters β_i, only the relative magnitudes of γ_1 and γ_2 matter, due to the normalization of the network term. Therefore, the sensitivity analysis mainly reflects the network component in the $S \to I$ dynamics. We understand that our text contained a potential source of confusion: whereas the network's role in the $S \to I$ dynamics is to actually model mobility of infected individuals across borders, network interconnections affect the dynamics of parameters β_i in a more abstract way, which is not a "mobility effect". In line with the Reviewer's comment, we have edited the revised manuscript to ensure we consistently denote it as a "network effect" instead, also where we discuss the benefit of including mobility data and, in particular, in the first Results-subsection and in the Discussion.

Finally, we note that the population's susceptibility to a new viral strain is precisely the type of uncertainty that can be modeled by the time-varying parameters β_i, which in our method are among the state variables estimated by the EKF. Parameters β_i are not known before a new epidemic wave starts, but they quickly adapt to observations thanks to the EKF; moreover, their dynamics are influenced by other countries through the network, efficiently spreading information about the new viral strain. To better convey this observation in the revised manuscript, we now mention before Eq. (7) the "varying transmissibility of different viral strains" as a factor influencing the dynamics of parameters β_i. We also mention such global effects as a motivation for including network effects in the dynamics of parameters β_i.

In addition, I believe that the EKF could benefit from enhancements:

- It would be useful to discuss whether the complexity of U is truly necessary for the filter's performance and for improving the results.

Response: Each diagonal element U_i of the diagonal matrix U consists of three components. First, the term modulated by ρ_1 necessarily arises from the assumption of independent detection of infections, which leads to a binomial distribution in the number of cases, and is the variance of the binomial distribution. Second, the constant term, as mentioned in the manuscript, is introduced to guarantee a lower bound for the variance during times of low incidence. Third,

the country-specific coefficient u_i, measuring the noise level in the country data stream, is directly estimated from the data without any optimization procedure; the noise in each country depends on factors that are unknown, such as the size of the sentinel networks used for data collection, or handling delays in regional data collection.

The constant variance component does not appear to be particularly impactful based on the sensitivity analysis for the network model, where both ρ_2 and the κ_2 parameter modulating the constant variance term in the state noise Q have quite small values. In the isolated models, however, these parameters have higher values, and play a fundamental role: in fact, if both parameters are set to zero, the covariance matrix S_t in the Kalman filter becomes (nearly) singular, which leads to an unstable filter. This does not happen in the network model, because the network propagates the uncertainty everywhere; therefore, even if in some country the incidence is nearly zero for a long time, the error variance does not converge to zero thanks to the network connections to other countries.

To address the Reviewer's comment, we have included in the revised manuscript a sentence that motivates the introduction of the country-specific noise scaling factors u_i right below their definition on p. 10, and we have added a discussion on the constant noise terms and the corresponding parameters κ_2 and ρ_2 into the last Result-subsection, along the lines of this response.

- σ_β is fixed. Its chosen value is not explained, and we do not know its sensitivity. This parameter directly affects the process noise covariance matrix Q (as shown in Eq. 8). From my experience with Kalman filters, adjusting Q can have a significant impact on the filter's gain and, consequently, on the correction of state estimates. A larger value of Q leads to more large corrections of predicted state values, while a smaller value results in more conservative corrections. In my view, it would be beneficial to allow σ_β be adjusted alongside other parameters during the simulated annealing.

- Page 13: Sorry I don't understand the role of K.

[This remark has been moved here, since it is very closely related to the previous remark. We will respond to both remarks here.]

Response: Indeed, adjustments of both Q and U have an impact on the KF sensitivity. However, if all covariance terms (Q, U, and the initial uncertainty P_0) are scaled by any positive constant, the Kalman gain does not change, and, consequentially, neither do the KF state estimates nor the noise free projections. Since the parameter fitting is based on these noise free projections, it is not possible to include all covariance-related parameters as tuning parameters. In other words, we wish to fit parameters by minimizing a cost function J that satisfies

$$\begin{aligned} &J(\gamma_1, \gamma_2, \sigma_\beta, \kappa_1, \kappa_2, \rho_1, \rho_2, k_1, k_2, k_3, \alpha_1, \alpha_2) \\ &= J(\gamma_1, \gamma_2, K^2\sigma_\beta, K^2\kappa_1, K^2\kappa_2, K^2\rho_1, K^2\rho_2, k_1, k_2, k_3, \alpha_1, \alpha_2) \end{aligned}$$

for any non-zero K^2 (scaling by K^2 is made to align with the manuscript's presentation). Identifying all covariance-related parameters at once can not be obtained by minimizing such a cost function, since they can only be obtained up to constant multiplication. To make the parameters identifiable, we fix σ_β during the parameter fitting procedure. It is true that increasing σ_β would result in larger KF updates to the β_i-parameters. However, precisely the same effect is achieved by decreasing κ_1, κ_2, ρ_1, and ρ_2. So, fixing σ_β is not a restriction in the generality of the Kalman gains, as only the relative magnitudes of these parameters matter.

In the subsequent noise calibration step, we set K by inspecting the coverage plots of probabilistic forecasts (Supplementary Fig. 5), for which K has a crucial role. As discussed, the chosen value for σ_β does not matter from the point of view of the KF state estimation. One possible choice would be simply to fix $\sigma_\beta = 1$ in the parameter fitting stage, in which case the scaling constant K^2 would directly have the interpretation of σ_β. However, to keep all parameter values at reasonable ranges, it is better to fix σ_β to a value that is deemed reasonable, that is, one that reflects our expectation on the changes of the β_i-parameters in given time unit. With such a choice, we would expect K to not deviate much from 1 (in terms of orders of magnitude). Having a reasonable choice for σ_β in the parameter fitting stage thereby helps evaluating the sanity of the calibration results. Originally, we used a value inherited from our earlier work, which was known to have a reasonable order of magnitude; however, such value was hard to interpret. To have a better explainable value, we have now rescaled σ_β to $\Delta t \mu^2$, where $\Delta t = 1/7$ (and all other covariance-related parameters accordingly). This choice means that in one week, one standard deviation of change in the β_i-parameters would correspond to the basic reproduction number $R_0 = \beta/\mu$ changing by one. As explained, this re-scaling has no effect on the results. However, the calibration parameter K is rescaled, and we have therefore re-defined the grid of values tried.

To address the Reviewer's comment, according to our response above, we have thus edited the manuscript at the end of the "Parameter fitting" section and at the beginning of the "Noise calibration for probabilistic forecasts" section to better clarify the need for the calibration step. We also explicitly point out that fixing σ_β is not a restriction for the parameter fitting approach to tune the KF sensitivity. We have added a new Supplementary note 3, where the equivalence of the KF state estimates for differently scaled covariances is proven. In addition, we explain the choice $\sigma_\beta = \Delta t \mu^2$ and its role in judging the sanity of the calibration results in the "Noise calibration for probabilistic forecasts" section.

Other- Details:

Page 6: the N_i are never defined.

Response: We thank the Reviewer for pointing this out. The definition was previously given after the first instance where N_i appears. We have now defined N_i as the population of country i on p. 6, where it first appears, after Eq. 2.

Page 7: References are needed for the choice of $\mu = 0.06$ and $\varphi = 0.0116$.

Response: Explanations about the choice of these parameters are now provided on p. 7. In particular, parameter μ is a small number, chosen so as to guarantee stability of state estimation by keeping the model's time scale at the same level as the data collection (one week). Parameter φ corresponds to immunity half-life of 60 days, which allows even re-infections during one epidemic season. ILI can be caused by different viruses, and therefore this is an effective parameter with reasonable magnitude.

Page 10: $T_{\max}$ not defined

Response: $T_{\max}$ has been replaced by 499, and we now mention on p. 10 that the average is taken over the entire dataset of 499 weeks.

Page 18: "The Kalman updates are therefore mainly focused on S and I, while the updates of β are fairly small.": OK but it's also depend on the values in Q, including σ_β.

Response: The Reviewer is right: the numerical value of κ_1 alone does not really allow to draw this conclusion. As this statement was mainly based on intuition and was not fully supported by the results, we have removed it from the text.

Page 18: Nice paragraph on identifiability, congratulations.

Response: We thank the Reviewer for the appreciation.

Page 20: "It can be observed (Fig. 5a and Supplementary Fig. 9) that the performance is on average slightly better for countries with larger population than with smaller population, but the performance improvement of the network model compared to isolated models is generally smaller for larger populations. . . . ": another possibility to consider is that random components (Q and U) may not always be a suitable fit for different demographics.

Response: To address the Reviewer's comment, first of all, we have noted in the revised manuscript, on p. 21, that the observed positive correlation between forecasting performance and the population size is entirely driven by the good performance for Italy and Spain (as mentioned in the caption of Supplementary Fig. 10a – formerly 9a).

To investigate whether the population size could give rise to different optima for the parameters, we have run an additional sensitivity analysis where different parameters (or parameter groups) were varied 10% up and down and country-specific performances were inspected; their differences were then plotted against the populations. These results are included in the new Supplementary Fig. 9. The only parameters where a mild trend ($R^2 = 0.066$ for the linear correlation) between population size and the performance gradient emerges are the ρ-parameters (controlling the measurement noise variance). Increasing these parameters for larger countries could improve performance, although overall the sensitivity to ρ-parameters is quite small. In addition, we summed up the absolute values of these performance gradients with respect to different parameters (Supplementary Fig. 9g). These values can be interpreted as "room for improvement" for each country if country-specific parameters would be fitted. Also in this plot, a small positive correlation emerges with population size, meaning that the fitted parameters in fact seem to fit better smaller countries (consistently with the observation in the main manuscript that the performance improvement of the network model is generally smaller for larger populations, and larger for smaller populations).

We now mention in the Discussion section the fact that the correlation between population and performance is due to Italy and Spain. We also acknowledge in the Discussion that country-specific parameter tuning might lead to better performance and refer to the new Supplementary Figure.

Reviewer #2 (Remarks to the Author):

I have carefully reviewed the revised version of Alto et al's manuscript. While I remain somewhat unconvinced by the authors' interpretation of the role of mobility, I find that arguments not directly supported by the content of the manuscript, such as the statement that "our network model has been included in the RespiCast platform", are of limited relevance to the scientific contribution of the study.

That said, the authors have made substantial efforts to address the comments and improve the manuscript. In my view, the paper is now suitable for publication in Communications Medicine.

Response: We thank the reviewer for the overall assessment of suitability for publication. The statement mentioned by the reviewer appears in the previous response letter rather than the manuscript. Therefore, we have not made changes to the manuscript based on this comment. In the letter, we merely mentioned that the networked version also outperforms the isolated models in the later RespiCast seasons, providing an additional validation. This, however, is not included in the manuscript.
